# Genomic Heterogeneity of Pancreatic Ductal Adenocarcinoma and Its Clinical Impact

**DOI:** 10.3390/cancers13174451

**Published:** 2021-09-03

**Authors:** María Laura Gutiérrez, Luis Muñoz-Bellvís, Alberto Orfao

**Affiliations:** 1 Department of Medicine and Cytometry Service (NUCLEUS), Universidad de Salamanca, 37007 Salamanca, Spain; mlgutierrez@usal.es; 2 Cancer Research Center (IBMCC-CSIC/USAL), 37007 Salamanca, Spain; luismb@usal.es; 3 Institute of Biomedical Research of Salamanca (IBSAL), 37007 Salamanca, Spain; 4 Biomedical Research Networking Centre Consortium-CIBER-CIBERONC, 28029 Madrid, Spain; 5 Service of General and Gastrointestinal Surgery, University Hospital of Salamanca, 37007 Salamanca, Spain

**Keywords:** pancreatic ductal adenocarcinoma, genetic heterogeneity, genomic subtypes, prognosis, chemoresistance

## Abstract

**Simple Summary:**

Although much progress has been made in recent years in the clinical management of solid tumors, pancreatic ductal adenocarcinoma (PDAC) remains a malignancy with limited therapeutical options. Indeed, responses to standard chemotherapy and targeted therapies vary widely when administered to unselected patient populations. This is in part due to the heterogeneous and variable molecular profile of PDAC. Here, we review current knowledge about the genomic heterogeneity of PDAC and its impact on disease behavior, and treatment including the molecular mechanisms of chemoresistance.

**Abstract:**

Pancreatic ductal adenocarcinoma (PDAC) is one of the leading causes of cancer death due to limited advances in recent years in early diagnosis and personalized therapy capable of overcoming tumor resistance to chemotherapy. In the last decades, significant advances have been achieved in the identification of recurrent genetic and molecular alterations of PDAC including those involving the *KRAS*, *CDKN2A*, *SMAD4*, and *TP53* driver genes. Despite these common genetic traits, PDAC are highly heterogeneous tumors at both the inter- and intra-tumoral genomic level, which might contribute to distinct tumor behavior and response to therapy, with variable patient outcomes. Despite this, genetic and genomic data on PDAC has had a limited impact on the clinical management of patients. Integration of genomic data for classification of PDAC into clinically defined entities—i.e., classical vs. squamous subtypes of PDAC—leading to different treatment approaches has the potential for significantly improving patient outcomes. In this review, we summarize current knowledge about the most relevant genomic subtypes of PDAC including the impact of distinct patterns of intra-tumoral genomic heterogeneity on the classification and clinical and therapeutic management of PDAC.

## 1. Introduction

Pancreatic ductal adenocarcinoma (PDAC) is one of the most lethal types of cancer [1], which may become the second leading cause of cancer-related death in 2030 [1,2]. At present, consensus exists on the close association between delayed diagnosis, lack of effective therapies, and/or resistance of neoplastic cells to conventional chemotherapy and the poor outcome of PDAC [3,4]. Like in other types of cancer, in order to overcome the poor prognosis of PDAC, a better understanding of tumor genetic, genomic, and epigenome alterations emerges as a critical step.

In the last two decades, important advances have been achieved in the identification of recurrent genetic and molecular alterations in PDAC [5,6,7,8]. Thus, at present, it is well established that virtually all PDAC carry activating (point) mutations of *KRAS* (93% of tumors) [9,10] and inactivating alterations of the *CDKN2A/p16* (95%) [3], *TP53* (72–74%) [6,9], and *SMAD4/DPC4* (50–78%) genes [3,6,9,11], which progressively accumulate from early pancreatic pre-neoplastic lesions to late-stage metastatic disease [12]. Despite these common genetic traits, PDAC consists of a highly heterogeneous group of tumors that frequently carry complex genetic profiles with many different coexisting alterations [4,9,10,13], including alterations that have been directly associated with transformation to PDAC [3,5,12,14,15,16,17,18,19,20,21,22,23,24,25].

In addition to all recurrently altered genes and signaling pathways, other less frequently altered genes (≈10% of all PDAC) [5,6,7,9,10,25] have also been described in PDAC. These include those coding for regulators of axon guidance (*ROBO2, SLIT2*) [6,7], disruption of the G1/S checkpoint machinery (*TP53BP2*) [10], DNA damage repair (*ATM, PALB2, BRCA1, BRCA2, SF3B1*) [6,7,9,26], and mismatch repair (MMR) genes (*MLH1, MSH2*, and *MSH6*) [27,28], together with genes involved in chromatin modification (*KDM6A, RNF43, PBRM1, ADRB1*, *EPC1, ARID2, SETD2*, and the *ASCOM* complex members *MLL2* and *MLL3* [6,7,9,10,27,29], and genes involved in the TGFβ- (*SMAD3, TGFBR1, TFGBR2, ACVR1B*, and *ACVR2A*) [5,6,7,9,10,27], MAPK- (*RREB1*, *MAP2K4*) [5,6,9] and PI3K/AKT/mTOR- (*PREX2*) [6] signaling pathways. Of note, some of these latter genetic alterations are frequently detected in PDAC patients with a genetic predisposition, particularly those involving germinal mutations in DNA damage repair genes and MMR genes (<15% of all hereditary PDAC tumors) [30].

In contrast to other solid tumors, such as breast cancer, lung cancer, and prostate cancer, where clinical and molecular classification systems have been adopted for better patient management, the clinical and prognostic impact of tumor genetics in PDAC still remains under investigation. This may be related, at least in part, to the lower prevalence and the great genetic heterogeneity of PDAC among different tumors [4] (and between as different cells inside a tumor) [3], together with its dismal outcome. In addition, current knowledge indicates that the clinical and biological behavior of a neoplasm depends not only on the underlying genetic alterations but also on the interaction between tumor cells and the surrounding tumor microenvironment (TME). In this regard, PDAC is a unique tumor in which neoplastic cells are surrounded by an abundant and dense stroma associated with tissue inflammation [31]. Thus, the tumor-stromal compartment of PDAC may represent up to 90% of the whole tumor mass and consists of extensive fibrosis, reduced vascularization, a hypoxic environment with an associated (highly variable) immune cell infiltrate (TILs). The fibrotic tumor tissue (identified as tissue desmoplasia by histopathology) mainly consists of cancer-associated fibroblasts (CAFs), endothelial cells, and pancreatic stellate cells (PSCs) immersed in a highly variable extracellular matrix (ECM) of collagen, glycosaminoglycans, proteoglycans, and growth factors [31], that further enhance tumor heterogeneity.

In this review, we provide a comprehensive summary of currently available data on the genomic heterogeneity of primary PDAC tumors at both the inter-tumoral and the intra-tumoral cell levels, and its potential implications on disease behavior and resistance to therapy.

## 2. Molecular Heterogeneity of PDAC

### 2.1. Genomic Signatures of PDAC Cells Associated with Patient Outcome

In the last decades, several attempts have been made to define molecular subgroups of PDAC with a significant impact on patient outcomes (Supplementary Table S1). This was facilitated by the progressive availability of a growing number of massive genome-wide analysis techniques, which provided the tools for the first attempts for an in-depth molecular characterization of PDAC.

Early studies evaluated the potential association between point mutations of the *KRAS*, *TP53*, *CDKN2A*, and *SMAD4* genes on the outcome of PDAC with contradictory results. Thus, Yachida et al. [32] established a direct relationship between the number of alterations in these genes and both disease free-survival (DFS) and overall survival (OS) of PDAC patients. Schlitter et al. [33] confirmed and extended these observations with the first proposal for a prognostic classification of PDAC based on histological, mutational, and DNA copy number alteration profiles. This included the definition of a poorer-prognostic subgroup of PDAC patients with adenosquamous or combined (gyriform, clear cell, papillary, and/or complex histological components) PDAC subtypes with *KRAS* mutations, altered *CDKN2A*, and >3 co-existing mutations of the *KRAS*, *TP53*, *CDKN2A*, and/or *SMAD4* genes [33]. In contrast, Dal Molin et al. [34] and others [35,36,37] found similar mutational profiles between long-term PDAC patient survivors (OS >10 years) and other PDAC patients, suggesting that these (and other) gene mutations on their own, might not provide sufficient prognostic discrimination in PDAC. In parallel, several studies have found an association between specific cytogenetic profiles identified by interphase fluorescence in situ hybridization (iFISH) and/or high-density single-nucleotide polymorphism (SNP) arrays and prognostic features of PDAC [13,38,39,40,41]. Thus, alterations of chromosomes 4, 7, 9q34 17q, and 20, in the absence of abnormalities involving chromosome 18q, including specific (numerical) alterations of chromosomes 4, 7, and 9q34 [11] and gains/amplification of chromosome 8q24, 17q, and 20q, have all been associated with more extended and disseminated disease at diagnosis and/or a poorer patient outcome [8,11,13,41,42,43].

In parallel, to these genetic studies, gene expression profiling (GEP) has also been extensively applied to define subgroups of PDAC patients with different clinical outcomes. Thus, Stratford et al. [44] proposed an algorithm based on overexpression of six genes (i.e., *FOSB*, *KLF6*, *NFKBIZ*, *ATP4A*, *GSG1*, and *SIGLEC11*) to identify PDAC patients at risk of developing metastatic disease. Likewise, Haider et al. [45], based on a meta-analysis, defined a 36-gene-based GEP associated with shorter survival and poorer outcome. This GEP signature included several genes (e.g., *ITGA5*, *KIF4A*, *CDC45*, and *NOSTRIN*) which had been previously identified to define GEP signatures related to the prognosis of PDAC [39,46], as well as some genes (*CDX2*, *CEBPA*, *SP1*, *STAT3*, *FOS*, *JUN*, and *BRCA1*) [47] which had even been validated at the protein level by immunohistochemistry. In a more recent study, a gene signature composed of 20 genes (PPS20) [48] was used to build a prognostic score that could discriminate among subgroups of PDAC patients with different outcomes and potentially distinct responses to targeted therapies. Thus, the PPS20 high-risk PDAC patient group is characterized by increased expression of proliferation-associated markers (CCNB, EGFR), DNA repair genes (RAD51), epithelial to mesenchymal transition (EMT) genes (EPS8) and cell motility, invasiveness, and tumor aggressiveness genes (TRIO, LDHA, MAP4K4, and ARNTL2); in contrast, low-risk PPS20 PDAC is characterized by a more differentiated pancreatic phenotype enriched in ion channel transport gene sets related to digestive enzyme secretion, together with genes involved in the inhibition of cell motility, migration and/or proliferation, and induction of cell cycle arrest (CBX7, MIA3, and KANK1), in association with a better patient outcome. Other poorer-prognosis GEP described for PDAC patients include those defined by a combination of genes involved in axonal guidance signaling pathways—the SLIT/ROBO pathway (*ROBO*, *ROBO3*) and semaphorins (*SEMA3A*, *PLXNA1*) [7,49]—and the p53/COX2 pathway, associated with upregulation of the miR-574-5p, miR-1244, and miR-474-5p miRNAs [50]. Other key components of cancer-associated signaling pathways, such as the *SMAD4* and *PIK3CA* genes also showed differential mutational profiles associated with upregulation of the TGF-β pathway in younger (<55 years) vs. older (≥70 years) PDAC patients, but they lacked prognostic relevance [51].

Despite all the above findings and prognostic associations, the genomic alterations described in PDAC in the above studies still remain unable to fully explain the heterogeneous clinical behavior of PDAC. Thus, further efforts have been dedicated to a more accurate definition of distinct molecular subtypes of PDAC with potential diagnostic and therapeutic implications via integration of genomic, transcriptomic, and/or epigenomic data.

### 2.2. Transcriptional Subtypes of PDAC

In 2011, Collisson et al. [46] defined for the first time three different subtypes of primary PDAC and human and murine cell lines, based on their transcriptomic profiles, with potential prognostic and therapeutic implications. These included the classical (35% of all PDAC), quasi-mesenchymal (30%), and exocrine-like (35%) subtypes of PDAC. Further independent studies confirmed and extended these findings with the identification of new molecular subtypes of PDAC which (completely or partially) overlap with those defined by Collisson et al. [46] (Supplementary Table S1). Despite all different nomenclatures and subtypes proposed so far, all classifications consistently share two molecular subtypes of PDAC with unique, recurrently different, profiles (Supplementary Table S1): (i) a well-differentiated subgroup of tumors defined by expression of epithelial genes named classical [9,35,46,52,53,54,55], GEP-A [24], pancreatic progenitor [10], epithelial [56], notch [57], lipogenic [58], non-glycolytic [59], and cholesterogenic [60] tumor subtype, which is associated with a better patient outcome; and, (ii) a poorly differentiated tumor subtype characterized by predominant expression of mesenchymal genes (named quasi-mesenchymal [46], GEP-B [24], squamous [9,10,55], basal-like [35,53,54], mesenchymal [56], hedgehog/Wnt [57], glycolytic [58,59,60], immune escape [36] and innate immune [61]), and a poorer patient outcome.

**Figure 1:**
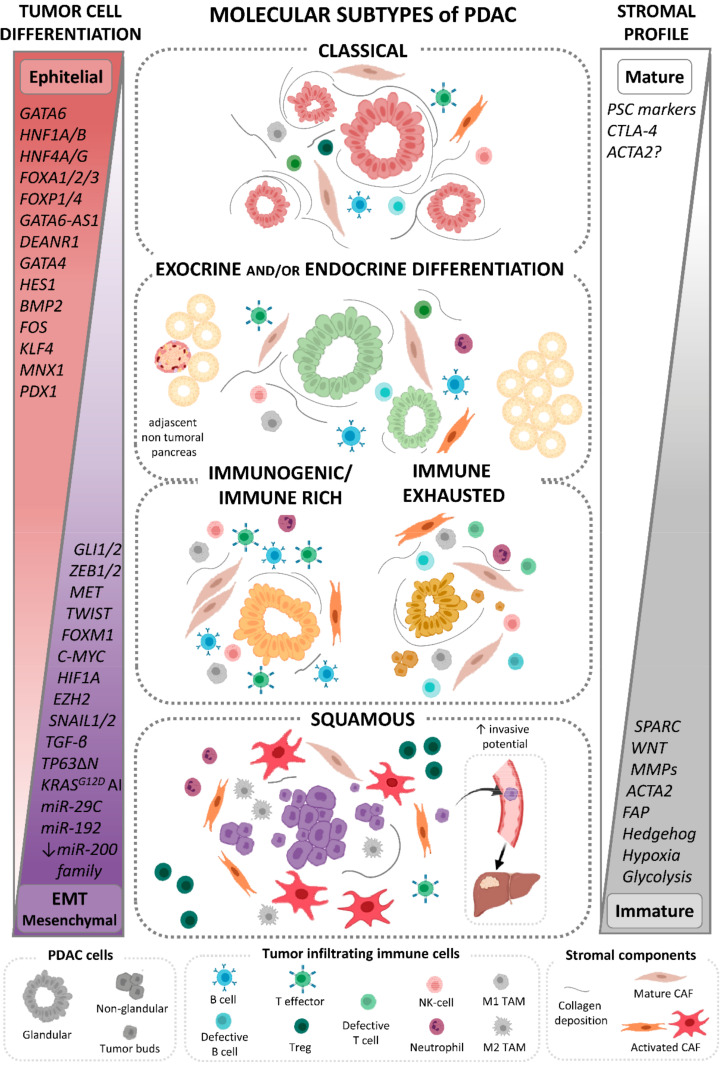
Key molecular, histopathological, and immunological features of the major molecular subtypes of PDAC defined based on the GEP of individual tumors. Classical, exocrine, and endocrine differentiated and immune-related PDAC harbor pancreatic epithelial cell differentiation driven by increased expression of endodermal identity genes (depicted in red), that are frequently associated with both low cellular collagen-rich (mature) stroma and variable profiles of tumor infiltrating immune cells. In contrast, squamous PDAC cells display histological dedifferentiation driven by the downregulation of pancreatic endodermal cell-fate determining genes and activation of EMT program (depicted in purple) to further acquisition of mesenchymal features, and are usually embedded in a highly cellular stroma enriched in activated CAFs (immature). EMT: epithelial-to-mesenchymal transition; PSC: pancreatic stellate cells; Treg: regulatory T lymphocytes; NK: natural killer cells; TAM: tumor-associated macrophages; CAF: cancer associated fibroblast; AI: allelic imbalance. (↓): decrease; (↑): increase. Created with BioRender.com.

In recent years, a consensus has been reached about these two PDAC tumor subtypes defined based on the analysis of neoplastic cells (i.e., isolated PDAC cells from laser capture microdissected tissues, cell lines, and/or orthotopic xenografts) and recognized now as classical and squamous PDAC according to the proposed harmonized nomenclature [23,62]. In the following sections, we describe the most relevant features of these two major classical and squamous PDAC subtypes. In addition, based on studies that used bulk tumor tissues with variable admixtures of tumor and stromal cells, other molecular subtypes of PDAC have also been identified which will be also discussed below (Figure 1; Supplementary Table S1).

#### 2.2.1. Classical Subtype of PDAC

The classical subtype of PDAC represents a major fraction (71%) of all PDAC [63]. It is characterized by an epithelial GEP consisting of an increased expression of epithelial markers, carcinoembrionary antigens, and cytokeratins (e.g., specific adhesion and epithelial genes such as E-cadherin and EpCAM) [24], together with a strong dependence of PDAC cells on the *KRAS* signaling pathway [46]. This main molecular signature of classical PDAC might be associated with the upregulation of several transcriptional programs involved in endodermal tissue identity and/or differentiation toward the pancreatic cell lineage. From the genetic point of view, it includes copy number gains and overexpression (associated or not with epigenetic activation) of *GATA6*, a transcription factor that maintains epithelial differentiation in PDAC cells [9,10,35,37,46,61,64,65,66,67,68], together with the up-regulation of *BMP2*, *FOS*, *FOXA1/2/3*, *FOXP1/4*, *GATA4*, *HES1*, *HNF4A/G*, *HNF1A/B*, *KLF4*, *MNX1*, *PDX1*, and *SHH* [10,37,65,66,67,69], and high expression of long non-coding RNA genes (lncRNAs) involved in pancreatic differentiation programs such as DEANR1 and GATA6-AS1 [9]. Remarkably, some of the above genes, such as HNF1A/B, HNF4A, and GATA6 [66,69,70] act as strong master regulators of PDAC development and thereby, they are also surrogate biomarkers for the classical subtype of PDAC [64].

Further transcriptional studies indicated that the classical PDAC GEP also involves a set of (altered) genes associated with cell proliferation, motility, tissue invasion, and tumor progression, together with high expression of mitochondrial components, ribosome, and angiogenesis signatures [24,56], in addition to genes involved in the innate and adaptive immune response, chronic immune pancreatic disease, cell stress, and tissue injury disease [24], in association with limited infiltration of the TME by TILs [61]. In turn, the classical PDAC gene expression program is strongly influenced by DNA and histone-based epigenetic regulators [54,67], leading to differential epigenomic landscapes that involve Ras signaling (e.g., KITLG, RASA3) and metabolic regulators (e.g., HKDC1, FBP1) [37] which appear to exert their regulatory influence on other transcription factors associated with the upregulation of lipid metabolism (PPARs) and major carcinogenic pathways [37]. In this regard, tumor cells from classical PDAC display cellular functions which are usually associated with other gastrointestinal tissues and that include pathways related to metabolite transport and fatty acid oxidation, steroid hormone biosynthesis and drug metabolism, whereas they retain some level of their typical pancreatic digestive function [54]. In line with these findings, unique metabolic features have also been reported among classical PDAC tumors [58,59,60] in association with a prominent metabolic adaptation [61] reflected by enrichment for lipogenic/cholesterogenic [58,59,60,63,68] or non-glycolytic [59,60] phenotypes. Thus, classical PDAC tumors show deregulation of mitochondrial pyruvate transport at the mRNA level leading to increased pyruvate shuttling into the mitochondria [60] that contributes to preferential use of glucose for the tricarboxylic acid cycle and an accelerated cholesterol uptake, and biosynthesis [58,68]. In turn, such lipogenic metabolic program is characterized by increased transcription of several glycerophospholipid genes involved in lipogenesis, sterol and cholesterol synthesis, and homeostasis [54,60,68], as well as in lipid and electron transport chain metabolite gene expression. In particular, overexpression of the NPC1L1 intestinal cholesterol uptake regulator (associated with extensive hypomethylation) [54,68] and cholesteryl ester occurs, supporting increased cholesterol absorption activity in classical PDAC [54]. Importantly, these metabolic features may be used for potential tailored treatment as some classical PDAC cell lines showed sensitivity to lipid inhibitors [58]. Despite this, evidence from patient samples and PDAC mice models revealed a metabolic trigger of the autocrine TGFβ signaling, the EMT, and thereby, of increased tumor aggressiveness, when cholesterol biosynthesis is reduced (e.g., by pharmacologic treatment with statins), which ultimately promotes a squamous phenotype associated with poorer patient outcomes [68].

The molecular background of classical PDAC translates at the histological level in the presence of a high proportion (>60%) of well-formed glands by morphology [71,72]. These include glands with a tubular stellate configuration, lined by pancreaticobiliary-type epithelium with well to moderate cytological atypia, or (usually large) tubulo-papillary glands with a wide range of sizes, consisting of (uniform) cells with low-grade dysplasia [72]. However, some specific subsets of classical PDAC such as the pancreatic progenitor tumors, are closely associated with the pancreaticobiliary subtype of intraductal papillary mucinous neoplasm (IPMN) and an invasive IPMN cancer histology [10], and they show moderately or poorly differentiated tumor cells [73]. Hence, classical PDAC typically consists of (low grade) well-differentiated tumor tissues [53,54,62], which frequently express (>10%) mucin markers associated with pancreatic differentiation [35], together with the O-linked glycosylated MUC5AC and MUC1 mucins, but not MUC2 or MUC6 [10].

#### 2.2.2. Squamous Subtype of PDAC

The squamous PDAC subtype is the most consistent PDAC phenotype across all classification systems proposed so far [23,74] and it represents between 17% and 54% of all PDAC [36,63]. In contrast to the classical subtype, squamous PDAC show decreased expression of epithelial markers, down-regulated expression of adhesion molecules, and genes involved in cell–cell adhesion-associated signaling pathways [24], together with upregulation of ECM related genes, and an inflammatory and strongly hypoxic GEP [10,27,68]. As the main characteristic of squamous PDAC, these tumors typically lack endodermal identity-associated genes due to down-regulation and/or hypermethylation of pancreatic endodermal cell-fate determining genes such as *GATA6*, *PDX1*, *MNX1*, *HNF1A/B* [9,10,67,69,75,76], and *HNF4A* [61,67], together with the *GATA6-AS1* and *LINC00261* lincRNAs non-coding genes [9,77] and/or expression of the *GATA6* transcriptional repressor EZH2 [75]. Consequently, deregulation of *GATA6* and *HNF1B*, loss of expression of the E-cadherin protein, and induction of EMT are all typical features of squamous PDAC that allow tumor cells to detach from the tumor mass and migrate [66,69]; in parallel, silencing of *GATA6* also increases the metastatic capacity of PDAC cells via direct inhibition of transcription factors such as FOXA1/2 [66].

From the genetic point of view, squamous PDAC frequently shows homozygous loss of *CDKN2A* [59,65], *TP53* deletion/mutation [9,10,59,65,71], *C-MYC* gain/amplification [78], and mutations of both the *SMAD4* and *PIK3CA* genes [36], together with recurrent mutations in key epigenetic regulator genes involved in chromatin modification (DNA methylation and acetylation) such as the *MLL2*, *MLL3* (also known as *KMT2D* and *KMT2C*, respectively) and *KDM6A* genes [10]. These findings highlight the potential relevance of several molecular pathways in this tumor subgroup, including those associated with chromatin modification and aberrant WNT signaling [6,54].

Even though all PDAC subtypes carry mutant *KRAS* as a dominant oncogene driver, greater transcript levels of mutant *KRAS*^G12D^ in association with EMT induction, upregulation of Vimentin, and repression of E-cadherin have been described in primary tumors [56,79] and human squamous (vs. other) PDAC cell lines [79]. Of note, overexpression of *KRAS*^G12D^ is frequently associated with amplification of the mutant *KRAS*^G12D^ allele relative to the wildtype allele [65,79] in both classical and squamous PDAC, but at significantly more pronounced ratios in the latter PDAC subtype [65] and in metastatic vs. primary tumors [65,79]. Altogether, these findings support a more aggressive, undifferentiated [59,79], and chemoresistant phenotype for tumors harboring a major *KRAS*^G12D^ allelic imbalance (compared to PDAC with no or minor *KRAS*^G12D^ imbalance) [65]; in addition, they point out a role for the *KRAS*^G12D^ dose in shaping PDAC cellular phenotypes, where a higher mutant *KRAS*^G12D^ dose may lead to increased Ras signaling and promotion of tumor metastases [65]. Interestingly, other genetic alterations typically observed in squamous PDAC, such as *TP53* deletion/mutation and loss of *CDKN2A*, also predisposed to more pronounced *KRAS*^G12D^ allelic imbalances in PDAC mouse models [79], supporting a role for this abnormality in shaping tumorigenesis in PDAC [79].

Altogether, the above genetic alterations lead to a unique downstream transcriptional response to oncogenic *KRAS* in squamous PDAC [35,79], which involve the Hedgehog/WNT (developmental) pathway [10,37,54,57,61,67], EGF [10,55], and other signaling pathways (PI3K-AKT and -mTOR, Hippo) associated with tumor aggressiveness [37,67,68], together with alterations in cell differentiation, proliferation and apoptosis-associated genes (e.g., *C-MYC*, *YAP1*, *HEY1*, and *E2F7*) [10,37,71,80].

On top of all the above, activation of other key transcriptional programs also converge in squamous PDAC in the development of mesenchymal features and increased tumor growth, aggressiveness, metastatic potential, and chemoresistance [10,58,60,67,80] of PDAC cells. Thus, activation of the TGF-β signaling pathway [65,67,68,81]—modulated by miR-29c and miR-192 [81]—leads to overexpression of primary drivers of a mesenchymal phenotype (ZEB1/2, TWIST, GLI1/2, and SNAI1/2) [10,24,37,57,65,68,69,80,82], while upregulation of the TP63ΔN transcriptional network in the presence of TP53 mutations [10,74], and hypermethylation of the MET receptor [37], regulate tumor cell plasticity and proliferation allowing for the development and establishment of a squamous PDAC transcriptional profile. Some of these molecular alterations found in squamous PDAC, such as inhibition of pancreatic epithelial differentiation genes (HNF4A and GATA6) [67] together with increased hypoxia [58,59,60,63,68], HIF1A, C-MYC [58,59,60] and WNT, insulin, and PI3K-AKT signaling [72], promote a metabolic rewiring of tumor cells to glycolysis. This is characterized by increased glucose uptake, ECM acidification (i.e., by prominent lactate production) and reduced oxygen consumption [67]. Accordingly, squamous PDAC more frequently present with tumor and stromal [54] copy number gains and/or increased expression of genes involved in glycolysis and the pentose phosphate signaling pathway (*LDHA*, *SLC16A3*, *TPI1*, *GAPDH*, *ENO1*, *LDHA*, and *PGK1*), regulation of glucose homeostasis (GSK3β) and hypoxia inducer genes (HIF1A and FOXM1) [67], along with downregulation of *NSDHL* and other cholesterol biosynthesis genes [68]. Once glycolysis is activated, induced expression of FOXM1 strengths the squamous PDAC phenotype by (over)stimulation of tumor hypoxia and the EMT [59]. These data suggest that induction of glycolysis together with inhibition of the cholesterol pathways in squamous PDAC may act as metabolic triggers for the EMT, whereas, once it is established, other factors (GLI1/2 and ZEB1) modulate lineage commitment, cell viability [68], and the acquired tumor resistance to chemotherapy [80]. These data highlight the strong correlation between EMT and the squamous PDAC gene expression program in this subtype of PDAC.

The major features of squamous PDAC described above (EMT, hypoxia, and glycolytic metabolism) all act together toward the development of unfavorable histological and immunological profiles [36,59,61,83,84,85]. Thus, histological dedifferentiation and increased capacity to dissociative growth and migration of squamous PDAC cells [36,72,84] is promoted by low expression of *HNF1A/B* genes [69,76] and miRNAs that regulate the EMT (miR-200a, miR-200b, miR-200c, miR-141, and miR-429) [86], together with increased—tumor and stromal—expression of the *SNAIL*, *ZEB1*, and *ZEB2* genes [86,87]. As a result, squamous PDAC usually displays poorly differentiated tissues phenotypes [53,54,62,73,79] including <40% of non-gland-forming ducts [72] associated with a squamous [71,72] and/or adenosquamous carcinoma-like morphology [10] with cells that lose adhesion and disconnect from the main tumor tissue on their own or as small groups of up to four cells [72], known as tumor buds, with unique features analogous to those of cancer stem cells (i.e., regarding drug resistance and metastatic potential) [85]; this may contribute to explain the poorer prognosis of PDAC tumors exhibiting high-grade tumor budding [36,84,87]. In turn, severe hypoxia and glycolysis promotes the suppression of anti-tumor immunity and enhancement of host immune evasion [36,59,83], while activation of the EMT-related Hedgehog signaling pathway activates CAFs to produce a dense ECM stromal deposition [57] which might exclude T cells from the squamous PDAC TME. These characteristics are reflected at the transcriptomics level on the absence of a specific immunogenic profile and increased expression of immunosuppressive pathways in squamous PDAC [24], confirmed in situ by low level immune cell infiltrates [36,53,61,74], deficient activation of B and T effector cells [36,59,83], and a relative enrichment on immunosuppressive cells (activated Tregs [36], M2-polarised macrophages [36,57,83,88], NK cells, neutrophils [53,61], and CTLA-4+ T leukocytes [83]). Since T effector cells are strongly dependent on aerobic glycolysis, the combination of high hypoxia levels and a glucose-depleted TME enriched in lactate production, observed in squamous PDAC, may further reinforce infiltrating T effector cells to rely on glycolysis, which would finally lead to their dysfunction [36,83,84]; in contrast, a significant increase in tumor-associated macrophages is observed in squamous PDAC, potentially due to their capacity of fatty acid oxidation to survive in a low-glucose concentration environment [83]. Finally, excessive lactate production by tumor cells and its release to the TME would also result in the inhibition of CD4+ T helper cells and NK cells, and activation and promotion of polarization towards an immunosuppressive phenotype of tumor infiltrating myeloid cells [59,89].

#### 2.2.3. PDAC Tumors with Exocrine and/or Endocrine Differentiation-Associated GEP

The third most frequently identified subtype of PDAC in the literature has been defined as exocrine-like [46], aberrantly differentiated endocrine-exocrine (ADEX) [10], secretory [56], notch [57], and quiescent [60] subtype of PDAC. It consists of tumors defined by transcriptional programs typically associated with terminally differentiated pancreatic tissue, characterized by upregulation of genes involved in exocrine (*NR5A2, MIST1*, *RBPJL*) and endocrine (*INS*, *NEUROD1*, *NKX2-2*) tissue differentiation, β-cell development, and tumor–derived digestive enzyme and pancreatic secretion (*CPA1, AMY2B, PRSS1*, *INS*) [10,35,56]. In addition to this GEP, exo/endocrine PDAC displays signs of activation of the notch signaling pathway [57] and reduced expression of genes involved in the amino acid catabolism, nucleotide metabolism, and pentose phosphate pathways, reflecting an overall low metabolic activity of these tumors. At the histopathological level, PDAC with exo/endocrine differentiation display specific features of rare acinar cell carcinomas [10], associated in some studies with a more prominent adaptive TILs and marked upregulation of genes known to play a role in immune checkpoint inhibition (e.g., *CTLA-4*, *BTLA, PD-1*, *TIGIT*) [57,61].

Although PDAC with exo/endocrine differentiation has been recurrently identified across independent studies, whether this subtype actually exists or results from contamination by adjacent normal tissue in low cellularity tumor samples—even after laser microdissection [9,35,52,53,55]—still remains a matter of discussion, suggesting that even small amounts of normal pancreatic tissue may disturb the output of the bulk tumor tissue transcriptome [9,52,53,54,55,90] (Figure 1). In this regard, it has been clearly demonstrated that sample purity influences cancer molecular subtyping. Thus, analysis of highly pure tumor cells is typically restricted to the classical and squamous subtypes of PDAC [9], while the exo/endocrine PDAC subtype is strongly associated with samples showing low tumor cell contents [9,71]. However, a unique methylation pattern has been reported in exo/endocrine PDAC vs normal pancreas, which mimics that observed in other PDAC [10]. These findings, together with the identification of exo/endocrine PDAC profiles in patient-derived xenografts, primary PDAC cell cultures [91], and organoids [92], suggest that tumor purity may not be the only determinant for this specific tumor subtype [10] and deserves further investigation.

#### 2.2.4. Immune-Related PDAC Subtypes

In several studies, other specific subtypes of PDAC have been identified which display distinct GEP related to immune cell function [61], TILs, and/or mechanisms for evading the host immune response [9,52,53,54,55,90] which range from immunogenic (anti-tumoral) to immunosuppressive (tolerant) tumor immune profiles associated with the morphologic appearance of the tumor and the behavior of the disease [84]. Thus, Bailey et al. [10] first reported on an immunogenic PDAC subtype enriched in immune signaling pathways, driven by a significant infiltration by leukocytes, with overlapping molecular and histological features with classical PDAC [10,36,53,55,73], but clearly distinct from the squamous, pancreatic progenitor and ADEX PDAC subtypes [10]. Such immunogenic PDAC subtype shows deregulation of B-cell function and antigen presentation along with up-regulation of toll-like receptor and CD4+ and CD8+ T cell-associated signaling pathways, including CD4+CD25+FOXP3+ Treg cell and immunosuppressive-associated features (CTLA-4 and PD-1) [10]. Subsequent studies confirmed these findings [53,59,61,73,93] and further identified new PDAC molecular subtypes that partially overlapped with the immunogenic and/or classical molecular profiles, that provide an additional layer of heterogeneity and complexity of the GEP of these tumors [36,53,57,61,73] which is potentially due to variable levels of immune cell infiltration in the tumor specimens [53,57,83]. Among these latter molecular signatures, the immune-rich PDAC [36] profile is defined by a high cytotoxic immune cell phenotype enriched in NK, B and T effector cells, M1-macrophages, and tertiary lymphoid tissue, along with reduced numbers of immunosuppressive cells (Tregs and M2-polarized macrophages), similarly to the immunogenic PDAC subtype. From the pathogenic point of view such heterogeneous immune profiles may be due, at least in part, to the different mutational burden, the number of tumor-associated neoantigens and the variable stromal components [54,59], as suggested by the fact that tumors with a low mutational burden are embedded in the reduced-volume immature stroma with low levels of expression of immune-associated markers, while PDAC with a higher mutational burden display greater amounts of TILs with highly variable immune cell subset contents [57,83]. Moreover, activation of specific immune evasion mechanisms in PDAC leads to an immune-exhausted PDAC phenotype (11% of all PDAC) [36] with shared features with immunogenic PDAC (i.e., immunogenic TME with lower levels of Treg cells, in association or not with deficient MMR and microsatellite instability; MSI) [10] associated with unfavorable immunosuppressive features such as upregulation of PD-L1, tumor budding, and an immune evasion phenotype, leading to a poorer biological tumor behavior similar to that of squamous PDAC [36].

Altogether, these findings illustrate the complexity of the immune cell microenvironment in PDAC and its contribution to tumor heterogeneity over a common classical PDAC molecular background [54], with potential implications for future immunotherapeutic strategies [83].

#### 2.2.5. Stromal Subtypes of PDAC

Increasing evidence suggests that the stroma of PDAC tumors is responsible, at least in part, for local tumor cell aggressiveness [4]. In contrast, the role of the tumor stroma during carcinogenesis remains controversial with both tumor growth supporting and restraining functions [94,95,96]. This is potentially due to the highly heterogeneous nature of the stromal components in PDAC and their relationship with neoplastic cells and some specific TME components (i.e., immune cells) [83]. In order to better address the evaluation of the role of the stroma on tumor growth and/or control, the characterization of the isolated tumor stromal compartment vs the bulk tumor has been pursued in several studies [35,62].

In a pilot study investigating the specific contribution of the tumor stroma to the overall molecular profile of PDAC, Moffitt et al. [35] identified two distinct stromal subtypes with prognostic consequences: the normal and activated stroma. These stroma profiles were found to be independent and complementary to the classical and basal-like PDAC tumor cell profiles simultaneously identified on individual tumors by these authors. Such observations have been further recapitulated, extended and refined in several transcriptomics [52,53,73,83,94,97] and proteomics [95] studies (Figure 1, Supplementary Table S1).

According to the classification proposed by Moffitt et al. [35], activated PDAC stroma tumors [35,53] (also known as immature [83], ACTA2-rich [97], FAP-rich [97], and ECM-rich [52] profiles), are characterized on histopathology by a small but highly cellular stroma tissue component with a limited collagen matrix and an overall immature stroma appearance [53,83,97]. It is typically associated with large and poorly differentiated tumors [97] and a more adverse patient outcome (i.e., inferior survival after tumor resection) [35,54,83,97,98]. From the molecular point of view, activated stroma tumors show overexpression of numerous ECM-associated genes involved in tumor promotion (*SPARC*) [35,53], hypoxia (*CA9*), glycolysis (*MCT4*) [83], the Hedgehog signaling pathway [53], and poor survival (*WNT2*, *WNT5A*, *MMP9*, and *MMP11*) [35]. This hypoxic and glycolytic phenotype of tumors carrying an activated stroma profile are associated with unique but heterogeneous immune infiltrates [35,61,99], and increased recruitment of immunosuppressive macrophages (*ITGAM*), chemokines (*CCL13*, *CCL18*), and T-cell phenotypes [35], with predominance of macrophage and peritumoral T cells enriched in CTLA-4+ and Treg lymphocytes [83] in some of the tumors vs. low CD8+ T-cell and intense neutrophil infiltration levels in other PDAC tumors with an activated stroma profile [97]. In this regard, more subsequent in-depth studies [97] further distinguished two subgroups of PDAC with an activated stroma profile: tumors with an ACTA2-dominant fibroblast-rich stroma (30% of PDAC) and PDAC neoplasms with an FAP-dominant fibroblast-rich stroma (44%), depending on the pattern of expression of the CAF activation markers ACTA2 (also known as α-SMA) and FAP, respectively [35,53,97]. Of note, the presence of FAP-expressing CAFs (previously related to poorer prognosis PDAC [100]) together with a high stroma activity and low collagen deposition, further confers an even poorer outcome among PDAC patients harboring an activated stroma profile [97,98].

In contrast to PDAC with an activated stroma profile, tumors with a normal PDAC stroma (26%) [97] (also known as mature [83], collagen-rich [97], desmoplastic [53], and immune-rich [52] tumors) display a tumor stroma with limited cellularity [83] which is enriched in collagen deposition (mature) around tumor glands [83,97,98], similarly to what is observed in chronic pancreatitis tissues [96], but with relatively higher expression of PSCs markers (i.e., VIM and DES) together [35] or not [97] with the expression of ACTA2. In addition, normal stroma PDAC show overexpression of CTLA-4 and vascular stromal components [53], together with downregulation of fibroblast marker genes (*FAP*, *PDPN*), metalloproteases (MMPs, ADAMs), and other ECM-associated genes (*FN1*, *POSTN*) [97]. As in the activated stroma subtype of PDAC, some degree of variability on the immune and inflammatory infiltrates is also observed within normal stroma PDAC; thus, while some tumors display relatively high levels of TILs (immune rich stroma) [52,53,54,73], scarce immune cell infiltrates associated with a pure normal stroma are observed in others tumors [83]. From the prognostic point of view, a normal stroma profile is associated with a better outcome [35], which is potentially due to the increased fibrogenic activity that may contribute to confine neoplastic cells, similarly to what happens in the healing phase of pancreatitis [98].

#### 2.2.6. Consensus Transcriptomics Subtypes of PDAC

Computational and/or physical removal of the stroma and stroma-associated GEP has contributed to a more refined (re-)classification of the molecular subtypes of PDAC [53,55]. Thus, genes used to define major subtypes of classical and squamous PDAC are heavily weighted toward the degree of epithelial differentiation (Figure 1), suggesting that these genes mostly provide information about the malignant tumor cell compartment, regardless of the (amount of) tumor stroma. In contrast, genes that define immune-related PDAC subtypes are mostly weighted toward the TME gene expression profiles, suggesting that in these molecular subtypes of PDAC the GEP identified are largely independent of the malignant tumor cell compartment [52,54,62].

Of note, despite that squamous and classical PDAC molecular profiles may be associated with both the activated and normal stroma patterns [10], a tendency towards a close relationship was observed between the tumor squamous PDAC and the activated stroma profiles on one side [52], and the classical PDAC and normal stroma patterns on the other side, with a continuous grading [52,54,97] in individual tumors (Figure 1). From the prognostic point of view, the tumor and stromal components act in a cumulative way [53,99] as compared to either compartment alone [52]. Thus, squamous PDAC with minimum or no stroma component was associated with the poorest prognosis, independently of stromal molecular phenotype [53]; in contrast, classical PDAC showed the best survival rates in the absence of (significant) stroma [101], while when embedded in an activated stroma profile classical PDAC had an intermediate prognosis [53]. Altogether, these data may contribute to explain, at least in part, the poorer clinical performance of stromal inhibitors targeting the, i.e., ACTA2 and Hedgehog pathways in PDAC preclinical trials in which reduction in tumor stroma was associated with poorer responses and increased tumor progression rates [102,103]. At the same time, these data also suggest that the stroma originating from surrounding host cells is closely associated with the tumor cell phenotype, which probably reflects a close interplay between the composition and function of TME and that of neoplastic cells (Figure 1).

**Figure 2:**
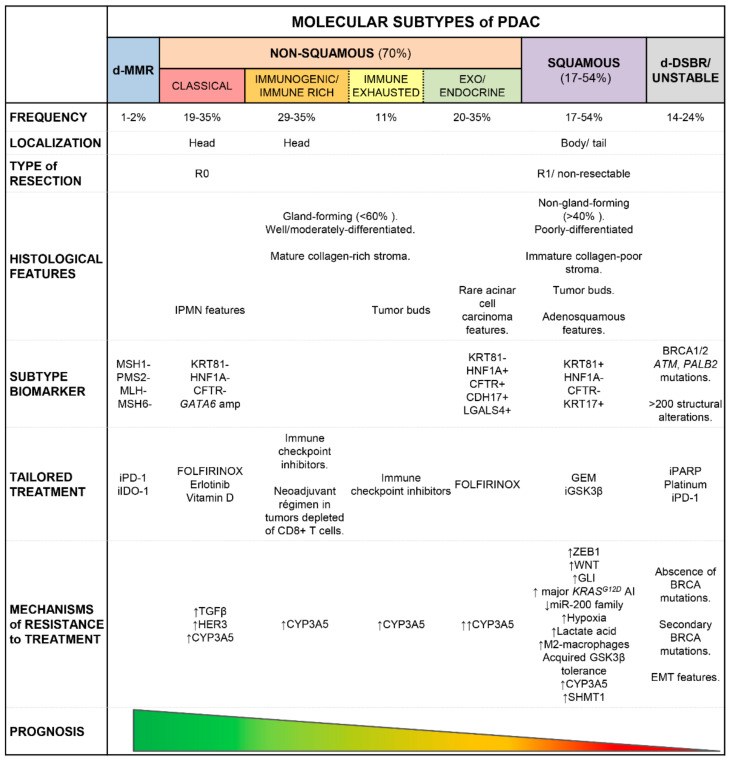
Schematic overview of the most relevant clinico-pathological features that have been recurrently associated with distinct molecular subtypes of PDAC. Dotted lines indicate non-well-defined clear-cut features between different molecular subtypes of PDAC. d-DSBR: double-strand break repair deficient; d-MMR: mismatch repair-deficient; GEM: gemcitabine; i: inhibitor; AI: allelic imbalance; amp: gene amplification; (↓): decrease; (↑): increase; (+): positive expression; (−): negative expression.

Based on all the above findings, it may be concluded that, as proposed by Collisson et al. [23], non-squamous PDAC would contain a spectrum of molecular subtypes of tumors that parallel the embryonic development of the pancreas, frequently embedded in a normal stroma frame which might be further subdivided into a classical-like or a more exo/endocrine differentiation-associated subtype of PDAC [52] (Figure 1). Subsequent subclassification of the above classical-like PDAC subtypes may consist of a pure classical PDAC and different immune-related subtypes of PDAC [9,52,53,54,55] (Figure 1 and Figure 2). In contrast, squamous PDAC are derived from the mesenchymal cell lineage defined by tumor cell-intrinsic features [56] and/or an admixture of epithelial tumor cell and activated stromal profiles [23] (Figure 1). However, more recent proteomics-based data also pointed out the existence of additional squamous PDAC subtypes including a proliferative and an inflammatory tumor subtype that requires further investigation and confirmatory studies [104].

## 3. Clinical Impact of the Distinct Molecular Subtypes of PDAC

### 3.1. Clinical Impact and Therapeutical Implications

As already indicated above, the molecular, as well as histological and morphological characteristics of PDAC, are all consistent with an association between the classical PDAC profiles and a better prognosis [27,46,53,57,60,61,62,68,69,72,105] compared to squamous PDAC [36,52,53,56,57,59,62,68,73,74] (Figure 2). Thus, classical PDAC have been more frequently associated with complete tumor resection [56], earlier stage (TNM stage I/II) disease [65] and prolonged overall survival (OS) rates ranging from 10.1 to 21.9 months [63]. In contrast, squamous PDAC would be associated with an earlier onset of the disease and larger tumors at (diagnostic) resection [59,74], together with higher rates (95%) of (unresectable) advanced disease [54,65,106,107], metastatic spread [37,53,74] and shorter DFS and OS rates [27,35,37,53,54,55,59,60,62,74,99,105,108] of 2 to 11.9 months [63,74] compared to classical PDAC, even when such comparisons are restricted to metastatic tumors [60,64,69]. In line with these observations, the squamous PDAC phenotype is significantly overrepresented among PDAC metastasis [27] (Figure 2). Despite all the above, in some studies nearly similar survival rates have been reported for classical vs. squamous PDAC [65,69].

In contrast to the clinical impact of the classical and squamous subtypes of PDAC described above, no consensus exists about the clinical significance of other well-differentiated (non-squamous) subtypes of PDAC [54,55] (Figure 2). Thus, while some reports suggest that PDAC tumors with an exo/endocrine differentiation (molecular) profile are associated with a better [57,61,91] or intermediate [46] clinical outcome (comparable to that of classical PDAC) [62], other studies reported a poorer survival for exo/endocrine differentiation tumors similar to that of squamous PDAC [56,69,109].

An even more complex scenario exists as regards patients harboring PDAC with distinct immune-related molecular profiles, with some reports suggesting that immunogenic and immune-rich PDAC profiles (Figure 2) could confer prolonged OS rates to PDAC patients [10,36] possibly due to the involvement of the adaptive immune response and higher rates of infiltrating CD8+ T cells in the TME, while those PDAC with an immune-exhausted PDAC profile (Figure 2) would be associated with a more adverse prognosis [36].

#### 3.1.1. Molecular Biomarkers for Risk Stratification of PDAC Patients

**Figure 3:**
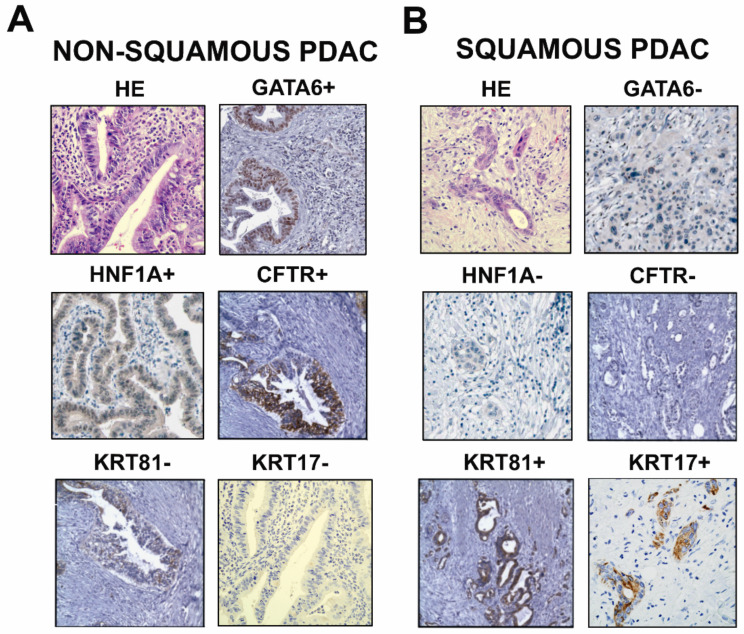
Detection of GATA6, HNF1A, CFTR, KRT81, and KRT17 PDAC molecular subtype-associated biomarker expression by immunohistochemistry of paraffin-embedded primary PDAC sections. Representative images of well/moderately differentiated tumor tissues displaying overexpression of GATA6 and lack of expression of KRT81 and KRT17, associated (i.e., exo/endocrine PDAC) or not (i.e., classical) with higher levels of HNF1A and CFTR proteins altogether are typically seen in tumors with non-squamous PDAC subtypes (**A**); whereas poorly-differentiated tissues with high expression levels of KRT81 and KRT17 showing no stain of GATA6, HNF1A, CFTR are frequently present in the squamous PDAC tumors (**B**). HE: hematoxylin and eosin staining; (+): positive expression; (−): negative expression. Images of GATA6 and HNF1A, and CFTR and KRT81 were obtained with permission from the Human Protein Atlas (v20.proteinatlas.org) and Henning et al., respectively [92].

Despite substantial progress has been made in the molecular subtyping of PDAC, there is still a lack of consensus on the specific genes and biomarkers that may be used for precise identification of the distinct molecular subtypes of PDAC. In order to overcome this limitation, several studies aimed at identifying combinations of GEP markers associated with specific molecular subtypes of PDAC have been developed, both in bulk resected tumors and in low-input (i.e., biopsy) samples, including formalin-fixed paraffin-embedded tumor tissues (Supplementary Table S1). Based on these studies, robust identification of PDAC patients harboring poor-prognosis squamous tumors and high-risk patients associated with poor treatment response may be reached by relatively simple nanostring- and/or PCR-based molecular classifiers such as the PurIST (Purity Independent Subtyping of Tumors) and ISP (Immune, Stromal and cell Proliferation signature) algorithms, which also facilitate the choice for more effective therapies in PDAC patients already at diagnosis [55], independently even of the neoadjuvant treatments that have been previously administered to the patients [99]. Further efforts directed to overcome limitations related to the effect of the mRNA quality decay on molecular subtyping of PDAC have been made in this regard (Figure 2 and Figure 3; Supplementary Table S1). Thus, the assessment of surrogate markers by immunohistochemistry such as KRT81 and HNF1A [91], CFTR [92], and/or CDH17 and LGALS4 [110], have been proposed for the identification of exocrine-like (HNF1A+, CFTR+, CDH17+ and/or LGALS4+ tumors), squamous (KRT81+), and classical (negative for all above markers) [91,105] PDAC (Figure 3). In addition to KRT81, staining for KRT17 also contributes to delineate tumors falling into the squamous PDAC [111] (Figure 3) and to identify tumors that are chemoresistant to gemcitabine- and 5-FU-based regimens [112]. Hence, simplified PDAC stratification by assessment of expression profiles for a restricted number of proteins provides the opportunity to define clinically relevant molecular subtypes of PDAC in routine diagnostic laboratories, in both resectable [92,105,113] and advanced-stage tumors [105] undergoing, e.g., volumetric whole-tumor analysis via radiomic-based investigations [113]. Further specific biomarkers remain to be established (at the protein level) for positive identification of classical (HNF1A-/CFTR- and KRT81-/KRT17-) PDAC.

#### 3.1.2. Therapeutic Implications of PDAC Transcriptomics Profiles

The reported prognostic advantage of classical PDAC has been particularly observed among patients treated with FOLFIRINOX (vs. gemcitabine-based) protocols [53,64]. This is even true when classical PDAC that display high levels of expression of the hENT1 nucleoside transporter are associated with an increased sensitivity to adjuvant gemcitabine therapy [114], or classical PDAC harboring liver metastases, are considered [104]. Other more recent treatment protocols that have been associated with a better in vitro response to chemotherapy of classical PDAC include combinations of inhibitors of MEK and EGFR (e.g., erlotinib) as well as HER3 [46,86], which enhance inhibition of cell proliferation and tumor growth, with a synergistic pro-apoptotic effect that sensitizes classical PDAC cells to chemotherapy [86].

In contrast, according to the COMPASS (Comprehensive Molecular Characterization of Advanced Pancreatic Ductal Adenocarcinoma for Better Treatment Selection, NCT02750657) trial [64] and other studies [59], squamous PDAC is a more aggressive tumor subtype that is less sensitive to first-line adjuvant chemotherapy based on FOLIFIRNOX [55,105], 5-FU, oxaliplatin [92], or isolated leucovorin [66], as well as combined (or not) MEK and EGFR inhibitors [46,86]. Overall, this may be due, at least in part, to alterations in drug delivery modulated by glycolysis [59], in addition to ZEB1-mediated resistance to MEK plus EGFR inhibitors [86]. Therefore, squamous PDAC is currently treated with conventional (adjuvant) gemcitabine-based protocols (in combination or not with oxaliplatin, irinotecan, or nab-paclitaxel) [46,57], particularly in case of locally advanced and metastatic disease [72], but with heterogeneous responses and frequent chemoresistance [56,65,113]. Resistance of squamous PDAC to gemcitabine is driven by differential upregulation on neoplastic cells of the WNT pathway [85,115,116] and enzymes such as SHMT1 [104]. In this regard, several stroma-dependent mechanisms have been associated with resistance to therapy in squamous PDAC such as those counteracting the cytotoxic effect of chemotherapy via activation of anti-apoptotic mechanisms, hypoxia-mediated resistance of EMT cells, and/or suppression of reactive oxygen species (ROS) by metabolic glycolysis, in addition to impaired drug metabolism, and decreased (passive) absorption of drugs by acidification of TME due to increased production of lactate and carbonic anhydrase by glycolysis and HIF1A-induced expression [85,117]. Acidification of the TME due to enhanced production of lactate also induces polarization of macrophages toward M2 immunosuppressive tumor-associated macrophages which further contribute to the development of resistance to gemcitabine via inhibition of caspase-3 mediated therapy-induced apoptosis [118] and decreased gemcitabine uptake by tumor cells associated with a release of pyrimidines [119], at the same time it enhances the development and maintenance of a squamous PDAC phenotype [59]. An alternative to standard chemotherapy regimens, promising data from pre-clinical studies has been reported on the potential benefit of the administration of inhibitors of the GSK3β (iGSK3β) regulator of glucose homeostasis associated with both the control of the glycolytic pathways that are altered in PDAC and sensitization of tumor cells to gemcitabine [67] in squamous PDAC tumors that express high levels of GSK3β. However, metabolic adaptation and tolerance to iGSK3β have been observed after iGSK3β-targeted monotherapy in a subset of squamous PDAC due to epigenetic activation of WNT ligands [67]. Based on these findings, future investigations are required to optimize treatment regimens based on iGSK3β (combined or not with chemotherapy), in which the effects of the drug on the transcriptomics and epigenetic profiles of PDAC are evaluated in parallel [67,120].

Neoplastic cells from exo/endocrine PDAC harbor an inherent ability to oxidize, metabolize, and inactivate small molecule drugs associated with increased CYP3A5 activity [91]. Thus, exo/endocrine PDAC might respond better to FOLFIRINOX [91,105] than gemcitabine, paclitaxel, and other small molecule drugs (i.e., erlotinib and dasatinib). Since CYP3A5 is not strictly required for normal cell homeostasis and it also mediates acquired drug resistance after longer-term chemotherapy in PDAC tumors subtypes other than the exo/endocrine subtype [91], the use of CYP3A5 inhibitors in combination with other subtype-specific tailored therapies (i.e., erlotinib in classical PDAC) emerges as a promising therapeutic option, not only for exo/endocrine but also for other molecular subtypes of PDAC [91].

Both the immunogenic (including immune-rich) and the immune-exhausted subtype of PDAC are currently considered good candidates for immune checkpoint blockade targeted therapies [36,121]. In the case of PDAC that lack tumor-infiltrating CD8+ T cells (i.e., immune-exclusion PDAC [36]), administration of immune checkpoint blockers might be combined with conventional chemotherapy (i.e., gemcitabine plus nab-paclitaxel) aimed at boosting the immune response via an increased CD8+ effector T-cell/FOXP3+ Treg ratio, associated with a significantly higher density of CD8+ T-cells inside (on-treatment) tumor samples, as observed among PDAC responders in phase II clinical trials (NCT02077881) [122]. Altogether, these data suggest that baseline assessment of the molecular profile and the cellular composition of TILs in treatment-naïve PDAC may contribute to better treatment decisions in PDAC patients.

Several clinical trials based on chemotherapeutic agents and novel targeted therapies, which have proven to be effective in other malignancies, have failed to benefit (unselected) PDAC patients. The few exceptions to this general rule include a small subgroup (7–8%) of PDAC patients that carry germline *BRCA1*, *BRCA2*, *ATM*, and *PALB2* mutations in whom combined treatment with cisplatin, other platinum compounds, mitomycin C, or poly(ADP-ribose) polymerase 1 (*PARP-1*) inhibitors (iPARP) have shown promising results [9,26,30,64,123]. Those findings have further led to the approval of the iPARP inhibitor olaparib for the treatment of PDAC by the US Food and Drug Administration (FDA) in 2019 [124]. In addition, Waddell et al. [6] and Connor et al. [26] in two pioneering studies on PDAC patients, identified an unstable vs. a stable genetic subtype of PDAC, as defined by the presence vs. absence of a large number of (structural) chromosomal gene rearrangements (>200) [6], including tumors carrying a double-strand break repair (DSBR) gene profile [26] characterized by either germline or somatic defects in genes involved in homologous recombination repair (HRD) of double-strand DNA breaks such as the *BRCA1*, *BRCA2*, *PALB2*, or *ATM* genes [6,26,64,125]. These tumor subtypes represent a subset of 14% and 24% of all PDAC patients which may benefit from therapeutic regimens based on agents that induce DNA damage (i.e., iPARP). However, still, around 10% of these latter PDAC patients did not show response to platinum-based chemotherapy protocols in clinical trials, which may be due to (i) the lack of germline or somatic inactivating mutations in *BRCA1/2* (“BRCAness” tumors) despite showing an HRD genomic profile [26], (ii) to common platinum resistance after secondary *BRCA1* or *BRCA2* mutations [126], (iii) upregulation of multidrug resistance transporters, and/or (iv) emerging EMT features in ATM-deficient neoplastic cells [127]. Altogether, these findings set the basis for the inclusion of genomic instability and the DSBR gene mutational profile genotypes in clinical trials (e.g., COMPASS [64]) based on platinum and/or novel drugs (e.g., iPARP) that target similar (DNA repair) mechanisms (Figure 2 and Supplementary Table S1).

In addition to the unstable/DSBR PDAC subtypes, another 1–2% of all PDAC display alterations that involve DNA mismatch repair (MMR) genes [26], as defined by unique MSH1, PMS2, MLH1, and MSH6 expression/mutational profiles found in primary PDAC tumors [36] and/or their paired metastasis [26] (Figure 2 and Supplementary Table S1). Although MMR contributes little to PDAC, patients bearing MMR signatures with MSI are less likely prone to be sensitive to 5-fluorouracil (5-FU) compared to other microsatellite-stable PDAC patients. Interestingly, PDAC tumors bearing DSBR and/or MMR gene signatures also exhibit a higher frequency of somatic mutations and tumor-associated neoantigens [128], which leads to increased immunogenicity associated with an enhanced local antitumor immunity, as reflected by increased activation of CD8+ T lymphocytes, a high T effector/Treg cell ratio, and overexpression of regulatory molecules such as CTLA-4, PD-1 and IDO-1 [36]. Altogether these findings suggest that assessment of markers that are potential candidates to be targeted by immunotherapy in DSBR and/or MMR PDAC might contribute to an improved rate of response to PD-1 or IDO-1 blockers such as pembrolizumab (approved by the FDA for first-line treatment of any solid tumor with dMMR and/or MSI [129]) in these specific subtypes of PDAC [26,130]. In contrast, these observations might also contribute to explain the limited success of immunotherapy in PDAC, beyond the dMMR and/or MSI genomic tumor subtypes.

Other potential therapeutic targets present in a small fraction of PDAC patients (1–2%) include genomic amplification of *ERBB2*, *MET*, *CDK6*, *PIK3CA*, and *PIK3R3* [6]. In this regard, it should be emphasized that early data from the COMPASS trial [64] revealed that 30% of PDAC patients harbor targetable somatic aberrations, such as mutations in the *ARID1A* and *PIK3CA* genes involved in activation of the PI3K pathway, that may respond to EZH2, ATR, and PARP inhibitors, as well as to distinct combinations of PI3K and CDK4/6 inhibitors; if this holds true, a significant subset of advanced PDAC patients might benefit in the future from such tailored therapies. However, in practice, the applicability of pharmacogenomics in PDAC currently remains largely restricted to the subgroup of PDAC patients that display unstable/DSBR and MMR genomic profiles.

### 3.2. Impact of Intratumoral Genomic Heterogeneity on the Molecular Subtype of PDAC

Although most PDAC may actually be classified as either classical or squamous tumors, in-depth molecular analyses have revealed that between 12% [63] and 27% [72] of PDAC display discordant transcriptional and/or morphological profiles which are compatible with a hybrid signature in which molecular features of classical and squamous PDAC coexist (Supplementary Table S1). Despite these findings possibly being related to a sampling bias (insufficient tumor areas analyzed to correctly define tumor morphology and/or preferential capture of a glandular component on microdissection prior to transcriptomics studies), a certain degree of intratumoral heterogeneity exists which might also contribute to explain such variability [56,95,131]. In order to assess the contribution of intra-tumor heterogeneity to the molecular subtyping of PDAC, Chan-Seng-Yue et al. [65] investigated the potential co-existence of squamous and classical cell populations at the single tumor cell level. Results from this study showed that such hybrid squamous/classical PDAC are much more frequent than expected (87% of all analyzed tumors) suggesting that in fact, tumors presenting with mixed classical/squamous cell features are due to the presence of more than one cell subpopulation within individual tumors expressing squamous vs classical tumor cell phenotypes [106]. These results were further confirmed and extended by others [56,60,65] who have even shown that such hybrid molecular subtypes also arise at the stromal cell compartment level [83]. In fact, single-cell transcriptomics studies of human PDAC specimens further this observation by highlighting the existence of up to four distinct subpopulations of neoplastic ductal cells present in all PDAC tumors but in different proportions [90,106], which display differential and/or overlapping gene expression programs (i.e., potential tumor progenitor; epithelial, classical, and/or proliferative; or squamous and/or invasive EMT ductal cells) [65,90,101,106,131,132], supporting the existence of a great level of inta-tumoral heterogeneity in PDAC [131]. This indicates that the molecular subtypes of PDAC cannot be fully recapitulated when individual (single) cell populations are analyzed at the intratumoral cell level vs. the bulk tumor.

Altogether, these findings highlight the fact that the great molecular heterogeneity of PDAC, may also be due to the lack of pure molecular profiles at the single-cell level within distinct cell populations that show different phenotypes and that are represented at variable proportions within individual tumors, intratumoral genomic heterogeneity thereby contributing to the final bulk tumor profile with clinical consequences [65,90,106,131]. Whether or not patients with discordant/hybrid tumor subtypes should be considered as a separate entity, rather than grouped into the major squamous or classical profiles, remains a matter of debate [63]. In this regard, a combinatory approach based on a squamous score (such as PurIST or ISP) in combination with surrogate biomarkers (e.g., HNF1A and KRT18) of specific molecular subtypes of PDAC [64] (Figure 3) will probably contribute to a more accurate and reproducible delineation between (unequivocal) classical and squamous tumors on one side, and PDAC patients showing less robust classical and squamous tumor signatures that cannot be directly assigned to either group [63]. Precise identification of PDAC patients harboring intermediate molecular features between the two well-defined tumor subtypes may be adopted in order to also predict for response to treatment and restrict specific cytotoxic drug therapies (i.e., FOLFIRINOX) to patients who will most likely benefit from them, avoiding its side effects in non-responding tumors such as those presenting hybrid molecular features between the two major subtypes of PDAC with a PurIST and/or ISP score close to the squamous PDAC phenotype [55,105,133].

### 3.3. Spatio-Temporal Heterogeneity of PDAC at the Molecular Level

The molecular features of primary PDAC also vary according to tumor localization in the pancreas, both at the macroscopic [108] and microscopic level [134,135]. Further to such spatial heterogeneity of PDAC, time adds another layer of complexity associated with both tumor cell plasticity and clonal selection [107,134] induced in part by therapy [136], together with changes in the TME [87,101]. Altogether, this raises an important issue in terms of the heterogeneity of a tumor depending on its localization and time of sampling, which should be considered, when assessing the molecular profile of PDAC, for its subsequent classification.

#### 3.3.1. Spatial Heterogeneity and Tumor Evolution

Spatial heterogeneity in PDAC is now well-established based on the reported association between the squamous [56,74,108] and the activated stroma [108] profiles, and tumor localization in the pancreas body and tail at diagnosis (Supplementary Table S1). These findings seem to reflect a relatively late clinical onset and molecular presentation of body/tail PDAC associated with ongoing genomic instability leading to advanced disease at diagnosis, which is ultimately associated with EMT and increased tumor cell proliferation [74,108]. Altogether, these and other features (e.g., larger and poorly-differentiated tumors) suggest that squamous PDAC would either present at later clinical stages of the disease [71] or it has an accelerated dedifferentiation pathway with a more aggressive biological behavior already at the earliest stages of the disease [74], compared to classical PDAC.

Based on the above findings, it has been hypothesized that classical PDAC would represent a baseline/default molecular subtype of PDAC, a subset of these tumors acquiring a squamous phenotype during tumor progression due to specific epigenomic changes that would occur during the evolution of these tumors [65,107]. In line with this hypothesis, a high frequency of classical PDAC in the pancreas head has been observed regardless of the tumor (clinical) stage, associated with high GATA6 transcript levels (typically detected in classical PDAC) in the benign pancreatic tissue adjacent to the tumor [65]. In addition, the existence of tumors that display a continuum of intermediate phenotypes between the classical and squamous subtypes, together with the coexistence of intra-tumoral tissue areas harboring neoplastic cell populations with a squamous phenotype within a glandular (classical) tumor background [71,107], and evidence of the switching of PDAC cells from a classical to a squamous tumor profile all point out a potential transition between the classical and squamous PDAC profiles during tumor evolution [63,105,113]. In line with this hypothesis, PDAC cells harboring genetic alterations which are typically associated with progression of PDAC (i.e., *C-MYC* copy number gain and gene amplification) [11] have been preferentially detected in tumor areas that display a squamous morphology when compared to those with a classical glandular appearance from the same tumor [71].

Another striking finding in PDAC localized in the pancreas body/tail is the lack of immunogenic tumor profiles with limited TIL [74,108]; these findings are in line with the close association reported between immunogenic and classical PDAC subtypes [23,36]. At the same time, they highlight a differential potential of distinct anatomic regions of the pancreas to enroll and activate immune cells (i.e., defective leukocyte recruitment to the distal regions of the pancreas) [108], with prognostic and therapeutic implications, as regards, e.g., immunotherapy.

In addition to the above-discussed variability observed among tumors localized in different anatomic regions of the pancreas, distinct molecular profiles and tumor cell populations have also been reported in response to adaptation to the TME within different areas of individual tumors such as the central area of the tumor vs. the invasive borders [137]. This includes distinct molecular profiles of both the single cells and the surrounding acellular components present in the different areas of individual tumors [101,135,138]. Altogether, these results suggest that neoplastic cells in PDAC rather, than behaving in a uniform way, may act as independent (tumor gland) units leading to differential responses to stress and a distinct predisposition to undergo EMT, proliferate, and invade surrounding tissues [90,101,135]. Indeed, recent single-cell RNAseq analysis demonstrated that tumor areas with low to moderate stromal content are frequently associated with EMT characteristics [90,101]. The variable proportion and localization of different populations of CAF cells (i.e., myofibroblasts, inflammatory, and MHC class II+ antigen-presenting cells) in the stroma surrounding the different areas of the tumor [94,132,139,140] may contribute to explain such heterogeneity of neoplastic cells among different tumor areas. These findings support the role of the stroma in shaping tumor architecture via modulation of the molecular profile and cellular composition of distinct tumor glands within individual PDAC tumors.

#### 3.3.2. Plasticity of PDAC Cells

In parallel to the influence of the stroma on the local tumor cell behavior, increasing evidence indicates that PDAC cells with epithelial (classical) and mesenchymal (squamous) molecular profiles have the ability to interconvert among the different phenotypic states [63,65] after chemotherapy [136], modulation of key molecular genes (*HNF4A*, *GATA6*, and *GLI1/2*) [56,67,141,142], in response to TGF-β [68,136] and/or under the influence of the TME due to re-shaping of the transcriptional programs of tumor cells [56,143]. Thus, treatment of PDAC cells with FOLFIRINOX [136] or autocrine activation of TGF-β (i.e., driven by forced *GLI2* expression, cholesterol-lowering statins, or loss of *HNF4A* and *GATA6* [67]) are sufficient to convert classical PDAC cells to squamous tumor cells [68,80]. Likewise, the switching of tumor cell transcriptomics profiles from squamous toward classical GEP has also been observed in different experimental models of PDAC via: (i) inhibition of *MET* [37] and *ZEB1* [86], (ii) blockade of *GLI2*, *OPN*, and molecular regulators derived from PSCs [80], and (iii) through depletion of CSF1R+ M2-macrophages (by targeting CSF1R or CXCR2) [88]. In contrast, administration of Vitamin D reinforces the epithelial phenotype of neoplastic PDAC cells with a baseline classical molecular profile, via reprogramming of CAFs into a more quiescent state in the absence of a clear shift of squamous tumor cells to a more classical phenotype [140]. Of note, evidence exists which indicates that interconversion of PDAC cells from a classical to a squamous phenotype might also occur spontaneously [65,80] in association with the acquisition of a major imbalance of *KRAS* during metastatic progression of a tumor, in the absence of other stimuli. However, changes from squamous PDAC toward a less aggressive (classical) disease phenotype might only be achieved when a tumor shifts from a major *KRAS* imbalance that disappears after therapy, due to subsequent outgrowth of minor clone(s) that carry no *KRAS* imbalance [136].

Altogether, these findings reinforce the dynamic nature of tumor gene expression and transcriptomics programs, underscoring the relevance of the TME vs. the tumor cells themselves in defining the molecular signatures of the tumor [88] and resistance to therapy via emergence and expansion of tumor subclones with different molecular alterations [134] and/or activation of distinct signaling pathways [78], that afford PDAC cells a different behavior and the potential to evade therapy, which ultimately determines disease behavior and patient outcome.

## 4. Conclusions

Overall, distinct genetic/molecular subtypes of PDAC exist which result from both inter-tumor and intra-tumor heterogeneity in tumor morphology and histopathology, cellular composition, and molecular profiles, all associated with a different clinical behavior, response to therapy, and patient outcome. To date, two major molecular profiles of PDAC (the classical and squamous subtypes) have been identified which are closely associated with unique stromal (e.g., normal mature, collagen-rich vs activated immature, collagen-poor stroma) patterns, with potential therapeutic implications. Whether or not these molecular profiles represent different stages of tumor evolution associated with EMT transition, from classical to squamous cell phenotypes, still remains to be fully demonstrated.

## Supplementary Materials

The following are available online at https://www.mdpi.com/article/10.3390/cancers13174451/s1, Table S1: Available genetic/molecular classifications of PDAC tumors.
