# Supplementary material for: Genomic Heterogeneity of Pancreatic Ductal Adenocarcinoma and Its Clinical Impact
Source: Cancers (Basel). 2021 Sep 3;13(17):4451. doi: 10.3390/cancers13174451 (PMC8430663; doi:10.3390/cancers13174451)
Supplement: Supplementary file 1 [file cancers-13-04451-s001.zip › cancers-1340538_R1_Supplementary Table 1.pdf]

Supplementary Materials

Genomic heterogeneity of pancreatic ductal adenocarcinoma and its clinical impact

María Laura Gutiérrez, Luis Muñoz-Bellvís and Alberto Orfao

Table S1: Genetic/molecular classifications of PDAC tumors proposed in the literature.

Disease features study	Type of analysis	Tissue analysis	N. of cases	Subtypes / signatures identified	Clinical relevance
Disease onset					
**Ben-Aharon *et al.* (2019)[1]**	Genomic + transcriptional	-	Publicly available data (MSK-IMPACT, TCGA-AU):<br>N=90 early-onset (≤ 55 years) + N=203 average-age onset (≥ 70 years)	Differential expression of SMAD4 (TGFβ) and PIK3CA signaling pathways and related proteins in early vs average age onset PDAC.	-
Histopathological features					
**Gutiérrez *et al.* (2011)[2]**	Genomic	Fresh-frozen macrodissected tumor	N=20	2 histopathological subtypes distinguished by CNA in 21 chromosome regions.	CNA signature differentiates smaller and well/moderately- vs. larger and poorly-differentiated tumors.
Prognosis					
**Stratford *at al.* (2010)[3]**	Transcriptional	Fresh-frozen macrodissected tumor	N= 15 metastatic vs. 15 local disease<br><br>Validation cohort: N=101 + N=50 (IHC)	6-gene signature: *FOSB, KLF6, NFKBIZ, ATP4A, GSG1, SIGLEC11.*	Prediction of occurrence of metastasis, prognosis and OS.
**Gutiérrez *et al.* (2011)[4]**	Cyto-molecular	Fresh-frozen bulk tumor	N=36	2 prognostic subtypes distinguished by CNA in 48 analyzed chromosome regions.	CNA in chromosomes 4, 6q, 7, 9p, 10q, 15, 17q, and 20, together with normal 18q associated with a more advanced disease at diagnosis; del(4p),CNA in 20p and nulisomy Y in males, associated with higher rates of death.

					Primary del(4p) defines a poor-prognosis clonal evolution pathway.
**Biankin *et al.* (2012)[5]**	Genomic	Fresh-frozen bulk or macrodissected tumor	N=142	-	SLIT-ROBO and semaforin pathways associated with a worse prognosis.
**Donahue *et al.* (2012)[6]**	Genomic + transcriptional	Fresh-frozen bulk tumor (> 30% tumor content)	N=25 (training) vs N= 7 non-T pancreatic tissue + N=42 (qRT-PCR) + N=148 (IHC) (validation)	171-integrated gene/miRNA/CNA.	Prediction of prognosis.
**Winter *et al.* (2012)[7]**	Transcriptional	Fresh-frozen bulk tumor	Training cohort: N=30 ()<br>Validation cohort: N=412 (IHC)	7-gene signature (*CDX2*, *CEBPA*, *SP1*, *STAT3*, *FOS*, *JUN*, *BRCA1*) for outcome prediction.<br><br>6-gene signature (*STAT3*, *FOS*, *JUN*, *CDX2*, *CEBPA*, *BRCA1*) for prognosis prediction after adjuvant therapy.	Prediction of prognosis and stratification of patients for adjuvant treatment.
**Yachida *et al.* (2012)[8]**	Mutational (*KRAS*, *CDKN2A* and *TP53* genes)	FFPE + Fresh-frozen bulk tumor	N=79	Stratification based on the number of mutated *KRAS*, *CDKN2A*, *TP53* and *SMAD4* genes in individual patients.	Correlated to patterns of disease progression, DFS, OS and metastatic failure.
**Gutiérrez *et al.* (2014)[9]**	Cyto-molecular	Fresh-frozen bulk tumor	N=55	3 prognostic subtypes distinguished by CNA in 3 chromosome regions.	CNA in chromosomes 4 and 9q34, and 8q24+ associated with a worse OS.<br><br>Construction of a prognostic cytogenetic score by combining these 3 chromosome regions.
**Haider *et al.* (2014)[10]**	Meta-analysis (transcriptional)	Meta-analysis	Training cohort: N=70<br><br>Validation cohort: N=246<br><br>Validation cohort: N=28 (gene-expression array + qRT-PCR)	36-gene signature.	Independent predictor of outcome.

**Nones *et al.* (2014)[11]**	Methylomic + transcriptional	Fresh-frozen bulk tumor	N=167 vs N=29 non-T pancreatic tissue<br><br>Validation cohort: N=121	-	Hypomethylation of *MET* and *ITGA2* (SLIT-ROBO signaling) associated with poorer survival.
**Dal Molin *et al.* (2015)[12]**	Genomic	Fresh-frozen macrodissected + FFPE microdissected	N=35 very-long term survival(>10 years)<br>Validation cohort: N=142	Mutational status of *BRAF, CDKN2A, GNAS, KRAS, PIK3CA, RNF43, SMAD4, TP53*, and *VHL*.	Mutational profile is not associated with a long-term survival.
**Namkung *et al.* (2016)[13]**	Transcriptional	Fresh-frozen bulk tumor	N=104	19-miRNA signature associated with the p53/COX2 pathway modulation; distinguish 3 molecular subgroups of PDAC.	6 miRNAs (miR-574-5p, miR-1244, miR-145-star, miR-328, miR-26b-star, and miR-4321) individually had a significant association with both OS and DFS<br><br>Upregulation of miR-574-5p, miR-1244, and miR-474-5p in the higher risk group of poor prognosis (OS and DFS) (20% of cases).
**Schlitter *et al.* (2017)[14]**	Mutational (*KRAS, CDKN2A* and *TP53*) + morphological + IHC	LCM FFPE	N=177	*KRAS, CDKN2A, TP53* and *SMAD4* mutational status + histologically subtypes defined 4 subtypes: conventional (51%), combined with a predominant component (41%), variant and special carcinomas (8%).	Worst OS associated with: adenosquamous and combined with complex component tumors + *KRAS*[mut] + altered *CDKN2A* + <3 mutations in *KRAS, CDKN2A, TP53* or *SMAD4.*
Treatment-based					
**Lowery *et al.* (2011)[15]**	Mutational (*BRCA1* and *BRCA2*)	N/A	N= 15 with a known *BRCA1* or *BRCA2* mutations	Identification of subgroups of patients carrying mutations in *BRCA1* and *BRCA2* genes.	Therapeutic benefit of combinations of poly(ADP-ribose) polymerase 1 (PARP-1) inhibitors and cisplatin, platinum compounds.

**Aung *et al.* (2018)[16]**	Genomic + transcriptional	LCM fresh-frozen biopsy	N=62 metastatic and locally advanced prior standard first-line therapy (COMPASS study)	PDAC tumors classified as:<br><br>unstable subtype (following Waddell´s), BRCA mutations and DSBR, other somatic genetic aberrations, basal-Like (24%) and classical (76%) (following Moffitt´s).	Stage III/IV PDAC patients with CL(M) tumors respond better to first-line chemotherapy compared to those with B-L tumors.<br><br>Better OS and DFS for CL(M) subgroup, specifically in those patients treated with modified FOLFIRINOX.<br><br>30% of patients harbor germline and somatic genetic aberrations benefits from tailored treatment.<br><br>10% of all PDAC would benefits from better to platinum-based chemotherapy (PARP inhibitors).<br><br>GATA6 expression is a robust surrogate biomarker for differentiating CL(M) and B-L subtypes.
Molecular PDAC subtyping					
**Collisson *et al.* (2011)[17]**	Transcriptional	FFPE LCM or macrodissected tumor	Discovery: N=27<br><br>Validation cohort: publicly available data (GSE15471,GSE16515);<br><br>Confirmation cohort: cell lines	62-gene signature (PDAssigner) of 3 PDAC subtypes: classical (35%), quasi-mesenchymal (30%) and exocrine-like (35%).	Q-M more sensitive to gemcitabine; CL more sensitive to erlotinib and has best prognosis.<br><br>CL displays overexpression of GATA6 + higher *KRAS* mRNA level and dependence on *KRAS*.
**Mirzoeva *et al.* (2013)[18]**	Genomic + transcriptional	-	N=29 cell lines	MEK + EGFR inhibitors results in strongly enhanced inhibition of cell and tumor growth independent of the *KRAS* mutation status.	Epithelial subtypes is sensitive for combinational effect of MEK + EGFR.<br><br>*ZEB1* expression was associated with resistance to the drug combination.
**Kim *et al.* (2014)[19]**	Transcriptional	Fresh-frozen bulk tumor	N=96	3 subtypes with impact in prognosis.	Subtype 2 and 3 has shorter OS; Subtype 2 showed distant metastasis.

			Validation cohort: publicly available data N=45 (GSE28735)		R0 resection rate was significantly higher in subtype 1.<br><br>Partial overlap with subtype 3 and E-L from Collisson.
**Daemen *et al.* (2015)[20]**	Metabolomic	PDAC cell lines	N=38 cell lines	3 subtypes: Slow proliferating (34%), glycolytic (27%) and lipogenic (39%).	Correlation with Collison´s:<br>glycolytic ≈ Q-M<br>lipogenic ≈ CL<br><br>Subtypes have influence on sensitivity of metabolic inhibitors.
**Galvan *et al.* (2015)[21]**	Histological + GEP (E-cadherin, β-catenin, SNAIL1, ZEB1, ZEB2, N-cadherin, TWIST1) + IHC	FFPE	N=120	Stromal subtypes associated with: tumor-budding, expression of EMT hallmarks, tumor and/or stromal cells expressing CDH1 repressors ZEB1, ZEB2 and SNAIL1.	Tumor-budding in TME phenotype associated with phenotypically aggressive PDAC.
**Gutiérrez *et al.* (2015)[22]**	Transcriptional	Fresh-frozen macrodissected tumor	N= 27 +<br>N=5 non-tumoral pancreas<br><br>Validation cohort: publicly available data (Collison)	2 subtypes: GEP-A and GEP-B	Overlaps:<br>GEP-A ≈ CL<br>GEP-B≈ Q-M
**Moffitt *et al.* (2015)[23]**	Genomic + transcriptional	Fresh-frozen bulk tumor<br><br>Virtual LCM	N= 145 + N=61 metastatic tumors + N=17 cell lines + N=46 non-tumoral pancreas + 88 distant site adjacent normal samples.<br><br>Validation cohort: N=15 + N= 37 PDXs + N=3 PDAC cell lines + N=6 CAF cell lines	2 PDAC subtypes: classical and basal-Like;<br>2 stromal subtypes: normal and activated	CL(M) subtype has strong overlap with the CL subtype defined by Collisson.<br><br>Stromal subtypes have influence on survival.<br><br>B-L subtype benefits from adjuvant treatment.
**Waddell *et al.* (2015)[24]**	Genomic	Fresh-frozen bulk tumor	N=100 +<br>N=25 PDCLs	4 genetic subtypes based on patterns of structural variations: stable (20%), locally rearranged (30%), scattered (36%), unstable (14%).	Unstable subtype associated with a high *BRCA* mutation signature, benefits from platinum treatment and PARP inhibitors.

**Bailey** ***et al.*** **(2016)[25]**	Genomic + transcriptional + methylomics	Fresh-frozen bulk tumor	N=342 + publicly available data N=74 + N=41 PDCLs	4 PDAC subtypes: squamous (31%), pancreatic progenitor (19%), immunogenic (29%) and aberrantly differentiated endocrine exocrine (21%; ADEX)	Partial overlaps with Collison: squamous ≈ Q-M ADEX ≈ E-L Pancreatic progenitor ≈ CL.<br><br>Upregulation of *CTLA-4* and *PD1* in immunogenic have therapeutic implications.
**Janky** ***et al.*** **(2016)[26]**	Transcriptional	Fresh-frozen bulk tumor	N=118 PDAC + N=13 non-tumoral pancreas	Confirmation of the PDAssigner gene signature.<br><br>3 subtypes: cluster k3.cl1, k3.cl2 and k3.cl3.<br><br>HNF1A/B are good candidates as master regulators of pancreatic differentiation	Overlap with Collison´s classification: k3.cl1 ≈CL k3.cl2 ≈ E-L k3.cl3≈Q-M<br><br>k3.cl1 and k3.cl3 associated with best survival; k3.cl2 with worst survival.
**Noll** ***et al.*** **(2016)[27]**	Transcriptional + mutational (*KRAS* and *TP53*)	PDXs + PACO cell lines	N=16 PDXs + N=8 cell lines.<br><br>Validation cohort: N=183 publicly available data datasets (TCGA) + N=231 (IHC)	Validation of Collison's subtypes: E-L(20%); CL/Q-M (80%).	Biomarkers identified: Q-M: KRT81+, HNF1A-, E-L: KRT81-, HNF1A+ CL: KRT81-, HNF1A-<br><br>CYP3A5 contributes to basal and acquired drug resistance and is a predictor for therapy response.<br><br>E-L tumors resistant to tyrosine kinase inhibitors and paclitaxel through *CYP3A5* upregulation.
**Birbaun** ***et al.*** **(2017)[28]**	Transcriptional	Bulk tumor + LCM (Collisson´s) + virtual LCM (Moffit´s)	Publicly available data: N= 846 (GSE15471, GSE42952, GSE28735, GSE55643, GSE43795, GSE36924, E-MEXP-2780, E-MEXP-950, TCGA-PAAD, Moffit´s, GSE12630, Bailey´s,	Validation of prognostic value of the Moffitt's, Collisson's and Bailey's classifications.	All classifications are associated with histopathological grade.<br><br>Confirmed the independent prognostic value of Moffitt tumor and stroma, and Bailey's classifications, but not that of the Collisson's classification.

			GSE57495, Collisson´s, GSE79670)		
**Connor** ***et al.*** **(2017)[29]**	Genomic + transcriptional	Discovery: fresh-frozen LCM<br><br>Replication: fresh-frozen bulk tumor	Discovery cohort: N=154<br><br>Replication cohort: N=95	4 main subtypes: age-related (70%), double stand break repair (11%; DSBR) mismatch repair (2%; MMR), unknown etiology.	DSBR partially overlaps with unstable subtype from Wadell´s and benefit from platinum treatment and PARP inhibitors.
**Knudsen** ***et al.*** **(2017)[30]**	Genomic+ transcriptional + IHC	FFPE	Discovery cohort: N=109 PDAC<br><br>Validation cohort: publicly available data N=183 (TCGA)	3 stromal subtypes: mature, immature and intermediate.<br><br>Immuno-composite classification (stromal + immune components + mutational) of PDAC in 4 subtypes.	Immature stroma, high levels of M2 TAM infiltration, CTLA-4+ lymphocytes and PDL1+ TME, are all correlated with shorter OS.
**Kuhlman** ***et al.*** **(2017)[31]**	Proteomic	-	N=12 PACO cell lines	Identification of Collison's subtypes biomarkers.	E-L: CDH17+, LGALS4+
**Nicolle** ***et al.*** **(2017)[32]**	Genomic + transcriptional + methylomic	Fresh frozen bulk tumor or biopsy	N=30 PDX (from 29 PDAC)	2 subtypes based on tumor and stroma: classical and basal-like. Classical tumors displays higher levels of NPC1L1 than basal-like.	All PDAC are sensitive to inhibition of NPC1L1 by Ezetimibe, but B-L PDAC are more sensitive to inhibition.
**Öhlund** ***et al.*** **(2017)[33]**	Transcriptional + proteomic + secretomic	PDAC tumors + co-cultures of murine and human PSCs and PDAC organoids	-	2 spatially separated, mutually exclusive, dynamic, and phenotypically distinct subtypes of CAFs: myofibroblast (myCAF) and-inflammatory fibroblast (iCAF).	Therapeutic development must consider intratumoral CAF heterogeneity to provide optimal benefits to PDAC patients.
**Raphael** ***et al.*** **(2017)[34]**	Genomic + transcriptional + methylomic + proteomic	Fresh-frozen bulk tumor	N=150 PDAC classified by: high purity cellularity (>33%) and low purity cellularity (<33%)	Confirmed 2 tumor-specific subtypes: basal-like/squamous and classical/pancreatic progenitor	High overlap with tumor classifications: high purity tumors≈ squamous/Q-M + progenitor/CL;<br>low purity tumors ≈ E-L/ADEX + immunogenic.<br><br>Favorable prognosis in low epithelial-mesenchymal transition and high MTOR pathway scores.

**Sivakumar *et al.* (2017)[35]**	Genomic + transcriptional	-	KRAS isogenic mouse ductal cell line + publicly available data N = 560 (ICGC, TCGA, GSE17891, GSE15471, GSE16515, GSE32676, GSE2109)	3 core regulating programs associated with previous PDAC subtypes: Hedgehog/WNT, notch and cell cycle.	Overlaps with Bailey´s classification: hedgehog/WNT ≈ squamous, notch≈ ADEX and pancreatic progenitor cell cycle ≈ immunogenic<br><br>Poor prognosis in hedgehog subtype and best prognosis for notch<br><br>Hedgehog and cell cycle subtypes are targets for immunotherapy, including subtype-specific checkpoint inhibition and myeloid depletion therapy.<br><br>Potential metabolic therapy in cell cycle subtype.
**Dreyer *et al.* (2018)[36]**	Transcriptional + genomic	-	Publicly available data N=518 (APGI)	Molecular distinction of head vs body/tail PDAC.	Worse prognostic of body/tail, association with squamous subtype and poor antitumor immune response.
**Erkan *et al.* (2018)[37]**	IHC	FFPE	N=233	Activated stroma index, ASI (area occupied by αSMA+ stromal cells/ area occupied by collagen).	ASI is an independent prognostic marker: high stromal activity (αSMA+) and low collagen deposition associated with a worse prognosis.
**Lomberk *et al.* (2018)[38]**	Genomic + epigenomic + transcriptional	Fresh-frozen bulk tumor	N=24 PDX (24 PDAC)	2 subtypes: classical and basal	Inactivation of *MET* results in a transition from a basal to more classical transcriptomic signature.<br><br>Potential therapeutic for inhibitors of highly upregulated molecules: EZH2 (tazemetostat), DNMT1 (decitabine), HDAC1 (vorinostat, trichostatin A).
**Muckenhuber *et al.* (2018)[39]**	IHC	FFPE	N=262 + N= 130<br><br>Validation cohort (non-resected):<br>N=64 (GEM-treated) + N=61 (FOLFIRINOX-treated)	Validation of Collison's subtypes biomarkers: KRT81 and HNF1A.	Worse prognosis of KRT81+ subtype.<br><br>KRT81+ subtype do not benefit from FOLFIRINOX.<br>HNF1A+ tumors benefits of FOLFIRINOX vs GEM -based treatment.

**Puleo *et al.* (2018)[40]**	Transcriptional + IHC + mutational	LCM or macrodissected FFPE	N=309<br><br>Validation cohort: N=269 (CGC + ICGC)	5 subtypes:<br>pure classical, immune classical, pure basal-like, stromal, activated and desmoplastic	Validation of B-L and CL subtypes.<br><br>Worst outcome for pure B-L subtype and pure CL and immune classical showed equivalent good prognosis.
**Wartenberg *et al.* (2018)[41]**	Mutational + IHC+ histopathological	FFPE	N=110	3 subtypes:<br>immune escape (54%); immune rich (35%); immune exhausted (11%).	Overlaps:<br>immune-scape ≈ Q-M/ squamous<br>immune-rich≈ pancreatic progenitor<br><br>Best outcomes in: low budding, low stromal FOXP3 counts, TLTs+, and *CDKN2A*^{wt}.
**Zhao *et al.* (2018)[42]**	Meta-analysis (transcriptional)	-	Publicly available data N=1200:<br>Training cohort (CGCarray, TCGA, MTAB-1791, GSE62165, GSE60980, GSE15471, GSE55643)<br><br>Validation cohort (Bailey, GSE71729, GSE57495 GSE79670, GSE62452, Collisson, GSE77858)	160-gene signature for subtype classification.<br><br>3 tumor-specific subtypes: L1 (22%), L2 (22%), L6 (23%)<br><br>3 stroma-specific subtypes: L3 (2%), L4 (14%), L5 (7%)	Worst clinical outcome for L2, poor survival for L3. Relative good survival for L4.<br><br>Partial overlaps:<br>L1, L6 ≈ CL(M);<br>L1, L3 ≈ CL;<br>L3 ≈ pancreatic progenitor;<br>L2 ≈ B-L/squamous/Q-M;<br>L1, L2, L6 ≈ activated stroma;<br>L6 ≈ ADEX/E-L;<br>L4 ≈ normal stroma;<br>L4, L1 ≈ immunogenic;
**Birbaun *et al.* (2019)[43]**	Transcriptional	-	Publicly available data N=249 (N=208 head vs N=41 body/tail; TCGA, Bailey's)	Molecular distinction of head vs body/tail PDAC.	Worse prognostic of body/tail.<br><br>Immunogenic subtype is more frequent in head tumors, and activated stroma subtype in body/tail tumors.
**Connor *et al.* (2019)[44]**	Genomic + transcriptional	Macrodissected, LCM or FACS-sorted fresh-frozen tissue or FFPE	N= 224 + N= 95 PDAC metastases	2 major subtypes: classical and basal-like.<br><br>50% of primary and metastatic PDAC characterized by hypoxia.	Moffitt´s subtyping significant for OS and PFS.<br><br>Partial response to therapy in tumors lacking hypoxia.
**de Santiago *et al.* (2019)[45]**	Meta-analysis (transcriptional + mutational)	-	Publicly available data: N= 242 (PACA-AU)	Consensus of Collisson´s, Bailey´s, Moffitt´s and Sivakumar	Overlaps:

			Validation cohort: N= 150 (TCGA) + N=120 (UNC)	classifications reveals 3 types of PDAC tumors, associated with 3 subtypes of TME: adaptive, innate and immune-exclusion immunologic signatures.	Type 1: innate immune ≈ B-L/ squamous/ Q-M/ activated stroma/ hedgehog (from Sivakumar)<br>Type 2: immunogenic (adaptive) ≈ ADEX/ E-L/ normal stroma/ notch (from Sivakumar).<br>Type 3: immune exclusion≈ CL and CL(M) subtypes)/ cell-cycle (from Sivakumar).<br><br>Worse prognosis for innate immune subtype.
**Dijik *et al.* (2020)[46]**	Transcriptional	Fresh-frozen tissues	N= 90<br><br>Validation cohort: Publicly available data (Bailey, PACA-AU and TCGA)<br><br>N=14 PDX +<br>N=9 cell lines	159-gene classifier distinguish 4 subgroups: epithelial, secretory, compound pancreatic and mesenchymal.	Poorer outcome for secretory and mesenchymal subtypes.<br><br>Overlaps:<br>secretory ≈ E-L/ADEX;<br>epithelial ≈ CL/ CL-M, pancreatic progenitor;<br>mesenchymal ≈ BL/ QM/ squamous<br><br>Compound pancreatic possibly arise from intratumoral heterogeneity with prominent mesenchymal profile.
**Elyada *et al.* (2019)[47]**	Transcriptional	Fresh-frozen human and mouse tissues + single cell	N=6+<br>N=2 paired non-tumoral pancreas	3 subclusters of tumoral ductal cells: two classical and one secretory.<br><br>Immune cells subpopulations: monocytes, macrophages (including alternatively activated macrophages), DC, NK cells, T CD4+ cells, Treg cells, exhausted T CD8+ cells.<br><br>3 CAF subpopulations: myofibroblast (myCAF), inflammatory fibroblast (iCAF)	Immunosuppression is mediated by immune cells + apCAFs.<br><br>apCAFs are potentially functionally or quantitatively modulated in response to immunotherapies.

				and antigen-presenting CAF (apCAF).	
**Follia *et al.* (2019)[48]**	Genomic + transcriptional + proteomic	-	Publicly available data:<br><br>Discovery cohort: N=176 (TCGA-PAAD)<br><br>Validation cohort: N=99 (ICGC)<br><br>Confirmation cohort: cell lines (CCLE) + N=31 serum from PDAC patients	2 glycolytic and 2 non-glycolytic subtypes.<br><br>Glycolitic: earlier disease, low immune-infiltrated tumors, depleted in CD4+ T cells, poor prognosis, and chromosome 12p13+ (associated with GAPDH, TPI1, and FOXM1 overexpression).	Overlaps:<br>glycolytic subtypes enriched in Q-M/ B-L/squamous;<br>Non-glycolytic subtypes enriched in E-L/ADEX and CL.<br><br>Poor prognosis in glycolytic.<br><br>Serum TPI1 levels is putative prognostic biomarker.
**Hening *et al.* (2019)[49]**	IF + qPCR	Organoids	Organoids from N=31 PDAC categorized in: Q-M, E-L and CL	Validation of KRT81 biomarker.<br><br>CFTR as a substitute for HNF1A for PDAC subtyping.	KRT81+ tumors are more resistant towards 5-FU and oxaliplatin.
**Kalimuthu *et al.* (2019)[50]**	Transcriptional + morphological	FFPE associated with transcriptomic data (Connor, 2017)	N=86	Pattern-based morphological classification reflects underlying tumor biology:<br><br>4 patterns:<br>conventional, tubule papillary, squamous and composite<br>+<br>2 subtypes based on the presence/absence of well-formed glands:<br>gland forming and non-gland forming.	Associations with Moffit´s profiles:<br>gland forming ≈ CL(M)<br>non-gland forming ≈ B-L<br><br>Presence >40% of non-gland forming associated with B-L and a worse prognosis.
**Ligorio *et al.* (2019)[51]**	Transcriptional + proteomic	Fresh-frozen tissue single cell + FFPE (RNA-ISH)	Co-culture of N= 6 cell lines + N=3 CAF PDCL.<br><br>Orthopic xenograft mouse<br><br>N=195 (TMA) + N=25 chemotherapy-treated	2 main phenotypes in PDAC cells: EMT and proliferative, PRO regulated by CAFs.<br><br>Primary tumors are composed of different tumor gland ‘‘units’’ each with distinct proliferative and metastatic propensity:	Stroma shapes single-cell and tumor gland heterogeneity<br><br>Predominantly EMT+PRO tumor glands associated with worsened patient survival<br><br>Importance of evaluating

				gland types EMT+PRO glands only enriched in high-stroma tumors, EMT glands enriched in medium-stroma tumors, and PRO glands in low-stroma tumors.	combination drug sensitivity with variations in stromal CAF composition
**Maurer *et al.* (2019)[52]**	Transcriptional + IHC	Fresh-frozen LCM (tumor epithelium and stroma compartments)	N=60 (tumor epithelium and stroma) + N=110 stromal PDAC<br><br>Validation cohort: Publicly available data N=350 (UNC, ICGC, PACA-AU, TCGA, PAAD)	Epithelium subtypes: basal-like and classical.<br><br>Stromal subtypes: immune–rich and ECM–rich.	Partial association between B-L epithelial and ECM-rich stromal compartments.<br><br>B-L + EMC-rich associated with a poor outcome.
**Neuzillet *et al.* (2019)[53]**	Transcriptional + IHC	Primary human CAF cultures + FFPE (IHC)	N=16<br><br>Publicly available data: N= 96 (Bailey *et al*)	4 CAF subtypes (A to D following pCAFassigner).	Overlaps:<br><br>Subtype A ≈ activated stroma<br>Subtype A ≈ QM and squamous<br><br>Subtype D-dominant had the poorest prognosis<br><br>Patients with C-dominant subtype had prolonged OS
**Rashid *et al.* (2019)[54]**	Meta-analysis (transcriptional)	Fresh-frozen bulk tumor + FFPE and FNA samples	Publicly available data:<br>Training cohort: N=321(Moffitt´s array, Aguirre and TCGA PAAD)<br>Validation cohort: N=378 (COMPASS, ICGC PACA-AU seq, ICGC PACA-AU array, Moffitt seq, Linehan seq, Connor)<br>+<br>Treatment cohort: N=68 (COMPASS, Linehan seq)<br>+<br>N=183 (bulk, FNA and FFPE samples)	2 subtypes by Purity Independent Subtyping of Tumors (PurIST) single-sample classifier: basal-like (related to Moffit´s) and classical (related to Collisson´s).	PurIST B-L tumors resistant to FOLIFIRNOX and worse OS.<br><br>Strong correspondence between:<br>B-L subtype ≈ squamous/ Q-M subtypes<br>CL(M) subtype ≈ remaining subtypes in the Bailey´s and Collisson´s.

**Brunton *et al.* (2020)[55]**	Genomic + transcriptional + epigenomic+ metabolomic+	PDAC PDCLs + GEMM	N=48+<br>Publicly available data:<br>N=342 (Bailey´s)	Identification of Bailey´s subtypes.<br><br>Plasticity between classical/pancreatic progenitor and squamous subtype regulated by *HNF4A* and *GATA6* expression.<br><br>*HNF4A* loss upregulates GSK3b and drives a squamous-like glycolytic profile.	Selective sensitivity of squamous subtype to pharmacological inhibition of GSK3β.<br><br>Identification of a subset of squamous PDAC with acquired GSK3β drug tolerance.
**Canli *et al.* (2020)[56]**	Transcriptional + Proteomic		Publicly available data:<br><br>Discovery cohort: N= 489 (PACA-CA, -AU, TCGA and GSE21501)<br><br>Validation cohort: N =66 + N=51 (GSE62452 and GSE79668)<br><br>Other used datasets: N=178 (TCGA-PAAD), N=51 (GSE79668), N=45 (GSE28735), N=123 (GSE71729),<br>N=37 PDAC cell lines (CCLE)	20-gene signature (Pancreatic cancer prognostic score 20, PPS20):<br>low PPS20 have higher TIL and CD8+/Treg cell rates;<br>high PPS20 associated with higher proliferation rates.	PPS20 relevant for discriminate prognostic, biological distinct subgroups of PDAC and prediction of response to targeted therapy.<br><br>Low PPS20 associated with a better OS and DFS.<br><br>Possible benefit of BIRB-796 drug (p38 MAPK inhibitor) for high PPS20 group, and Ouabain (inhibitor of the Na+/K +-ATPase) for low PPS20 group.
**Chan-Seng-Yue *et al.* (2020)[57]**	Mutational + transcriptional	LCM Fresh-frozen tissue + single-cell	N= 314 (N=248 with RNA-seq)	Basal-like-A, basal-like-B, hybrid, classical-A<br>and classical-B.<br><br>Classical-A/B tumors associated with homozygous *SMAD4* loss and *GATA6* amp; basal-like-A/B tumors associated with *TP53*^{mut}, complete loss of *CDKN2A* and *KRAS*^{mut} imbalance.	-

				Basal-like and classical expression signatures can exist intratumorally.	
**Glaβ *et al.* (2020)[58]**	Transcriptional		Publicly available data: N=177 (TCGA PAAD) + N=248 normal pancreas (GTEx) + cell lines (CCLE)	27-lincRNAs signature differentiates classical vs basal-like.	-
**Hayashi *et al.* (2020)[59]**	Histological + transcriptional + genomic	Fresh-frozen bulk tumor + FFPE +multiregional macrodissection	N=123 (FFPE)<br><br>N=23 + 93 (of 123 fresh-frozen primary and paired metastasis, respectively)<br><br>Publicly available data: N= 145 (TCGA) + N=617 (MSK-IMPACT)	Squamous histological morphology corresponds to basal-like/quasi-mesenchymal/squamous subtypes; glandular morphology to classical subtype.<br><br>Regions with squamous morphology and basal-like characteristics represent a subclonal population within a glandular tumor.	-
**Juiz *et al.* (2020)[60]**	Transcriptional	Biopsy-derived organoids + single-cell	N= 6 classical PDAC	4 cell subpopulations with possible differences in tumor aggressiveness.<br><br>Classical and basal-like cells coexist in the same tumor.	Overlaps:<br>C1 cluster ≈ B-L cells
**Kalloger *et al.* (2020)[61]**	Transcriptional (49-gene from Moffit)	FFPE LCM	N=48 short term survivors (>=4 and <12 months) + N=48 long term survivors (>3 years)	Validation of Moffit´s classification on epithelial and stromal compartments.	Stromal *LY6D* expression is protective.<br><br>Poor prognosis in tumors displaying decreased stromal LY6D, and increased stromal CTSV and epithelial KRT6A protein expression.
**Kandimalla *et al.* (2020)[62]**	Transcriptional	FFPE	Publicly available data Discovery cohort: N=163 (TCGA)	15-gene immune, stromal and proliferation (ISP) gene signature.	ISP-poor OS signature identify squamous/Q-M/B-L/pure B-L and stroma activated subtypes.<br><br>Risk-stratification using ISP signature, CA19-9 levels and T/N-stages.

			Validation cohort: N=506 (ICGC, E-MTAB-6134, GSE71729) + N=119 (q-RT-PCR)		
**Karasinska *et al.* (2020)[63]**	Genomic + transcriptional	.	Publicly available data: N= 325 (TCGA, ICGC, COMPASS, PanGen, POG).	Prognostic metabolic (glycolytic and cholesterogenic pathways) subgroups: quiescent (31.1%), mixed (24.3%), cholesterogenic (22.5%) and glycolytic (22.2%)	Glycolytic tumors are more aggressive (or less sensitive) to chemotherapy.<br><br>Overlaps:<br>quiescent ≈ CL/ ADEX /E-L<br>glycolytic ≈ B-L/ squamous/ Q-M<br>cholesterogenic ≈progenitor, CL-M
**Kassis *et al.* (2020)[64]**	Radiomic-based phenotyping + proteomic	CT-scan images + FFPE	N= 207 PDAC	Image-derived phenotypes representative of immunohistochemically (HNF1A and KRT81) defined molecular subtypes.	Clinically relevant phenotyping system to better inform precision therapy regimens.
**Lin *et al.* (2020)[65]**	Transcriptional	Fresh-froze tissue + single cell	N= 10 primary tumors + N=6 metastasis<br><br>Publicly available data: (TCGA -US, -CAN, -AU)	7 major cell populations: ETCs, EMTs, CAFs, DCs, Endos, TILs, and TAMs.<br><br>3 major cell populations in metastasis: ETCs, TILs, and TAMs High inter-patient heterogeneity of tumor cells.	Cellular composition of tumors defines PDAC subtypes and correlated with patient outcome.<br><br>High expression of tumor cell signature associated with a shorter OS.
**Ogawa *et al.* (2020)[56]**	IHC + histological + transcriptional	FFPE + fresh frozen tissue	N= 212 (IHC) + N= 20	3 subtype of stroma: collagen-rich stroma (C-stroma, 26%), FAP-dominant fibroblast-rich stroma (F-stroma, 44%); ACTA2-dominant fibroblast-rich stroma (A-stroma, 30%).	Overlaps:<br>C-stroma ≈ normal stroma (Moffitt),<br>A-stroma or F-stroma≈ activated stroma (Moffitt)<br>C-stroma≈ CL/CL(M)<br><br>A-stroma exhibited poorer prognosis and C-stroma best outcome.
**Tophan *et al.* (2020)[66]**	Transcriptional + genomic		Publicly available data: N=574 (TCGA PAAD-US, ICGC PACA-CA, PanGen, CT02869802, POG,	Analysis of Moffitt´s, Collisson´s, Bailey´s, and Karasinska´s consistency classifiers.	12% of tumors displays subtype-discordant showing intermediate survival (CL and B-L genes were concomitantly expressed and

			NCT02155621, COMPASS, NCT02750657)	Non-binary nature of PDAC subtypes.	intermediate mutant *KRAS* allelic imbalance).
**Zhang *et al.* (2020)[67]**	Transcriptional	-	Publicly available data:<br><br>Discovery cohort: N= 149 (TCGA)<br><br>Validation cohort: N=160 (Baileys, E-MTAB-6830) + N=12 cell lines (CCLE)	Squamous subtype influenced by miR-29c and miR-192 regulating TGFβ signaling pathway.	Strong association with prognosis miR-29c target genes *LOXL2*, *ADAM12* and *SERPINH1.*<br><br>LOXL2 and SERPINH1 high expression demonstrated high performance for predicting lower sensitivity of ZD-6474 (Saracatinib) drug.

ADEX: aberrantly differentiated endocrine exocrine PDAC subtype (following Bailey´s); APGI: Australian Pancreatic Cancer Genome Initiative; B-L: basal-like PDAC subtype (following Moffit´s tumor classification); CA19.9: carbohydrate associated antigen; CAF: cancer associated fibroblast; CCLE: Cancer Cell Line Encyclopedia; CGC: Cancer Genome Consortium; CL: classical PDAC subtype (following Collisson´s); CL(M): classical PDAC subtype (following Moffit´s tumor classification); CNA: copy number alteration; COMPASS: Comprehensive Molecular Characterization of Advanced Pancreatic Ductal Adenocarcinoma for Better Treatment Selection study; CT-scan: computed tomography; DC: dendritic cells; DFS: disease free survival; DSBR: double-strand break repair deficiency; E-L: exocrine-like PDAC subtype (following Collisson´s); ETC; epithelial tumor cells; FFPE: formalin fixed paraffin embedded; FNA: fine-needle aspiration; EMC: extracellular matrix associated; EMT: epithelial-mesenchymal transition; GEM: gemcitabine; GEMM: genetically engineered mouse model; GEP: gene expression profile; GTEX: Genotype-Tissue Expression project; HHR: homologous recombination repair; ICGC: International Cancer Genome Consortium; IF: immunofluorescence; IHC: immunohistochemistry; IPMN: intraductal papillary mucinous neoplasm; LCM: laser capture microdissection; LC-MS/MS, liquid-chromatography tandem-mass spectrometry; MHC-II: major histocompatibility complex class II; MMR: mismatch repair; MSK-IMPACT: Memorial Sloan Kettering-Integrated Mutation Profiling of Actionable Cancer Targets; MSI: microsatellite instability; N/A: data not available; NK: natural killer cells; Non-T: non-tumoral pancreatic tissues; OS: overall survival; PACO: propagate primary PDAC cell lines; PanGen: Prospectively Defining Metastatic Pancreatic Ductal Adenocarcinoma Subtypes by Comprehensive Genomic Analysis; PDAC: pancreatic ductal adenocarcinoma; PDCL: patient derived cell lines; PDX: patient-derived xenograft; PFS: progression free disease; POG: BC Cancer Personalized OncoGenomics program; Q-M: quasi-mesenchymal PDAC subtype (following Collisson´s); qRT-PCR: quantitative real time PCR; TAM: tumor associated macrophage; PSC: pancreatic stellate cell; TMA: tissue matrix arrays; TCGA: The Cancer Genome Atlas from Australia (-AU), Canada (-CA) or United States (-US) partners, and pancreatic adenocarcinoma (-PAAD) subproject; TIL: tumor infiltrating lymphocytes; TLT: Tertiary lymphoid tissue; T/N: tumor/ lymph node classification of malignant tumors staging, TNM; TME: tumor microenvironment; Treg: T regulatory cells; UNC: University of North Carolina Chapel Hill; 5-FU: 5-Fluorouracil.

References for Supplementary Table 1

1. Ben-Aharon, I.; Elkabets, M.; Pelossof, R.; Yu, K.H.; Iacubuzio-Donahue, C.A.; Leach, S.D.; Lowery, M.A.; Goodman, K.A.; O'Reilly, E.M. Genomic Landscape of Pancreatic Adenocarcinoma in Younger versus Older Patients: Does Age Matter? *Clin Cancer Res* **2019**, *25*, 2185-2193, doi:10.1158/1078-0432.CCR-18-3042.
2. Gutierrez, M.L.; Munoz-Bellvis, L.; Abad, M.M.; Bengoechea, O.; Gonzalez-Gonzalez, M.; Orfao, A.; Sayagues, J.M. Association between genetic subgroups of pancreatic ductal adenocarcinoma defined by high density 500 K SNP-arrays and tumor histopathology. *PLoS.One.* **2011**, *6*, e22315.
3. Stratford, J.K.; Bentrem, D.J.; Anderson, J.M.; Fan, C.; Volmar, K.A.; Marron, J.S.; Routh, E.D.; Caskey, L.S.; Samuel, J.C.; Der, C.J.; et al. A six-gene signature predicts survival of patients with localized pancreatic ductal adenocarcinoma. *PLoS Med* **2010**, *7*, e1000307, doi:10.1371/journal.pmed.1000307.
4. Gutierrez, M.L.; Sayagues, J.M.; Abad, M.M.; Bengoechea, O.; Gonzalez-Gonzalez, M.; Orfao, A.; Munoz-Bellvis, L. Cytogenetic heterogeneity of pancreatic ductal adenocarcinomas: identification of intratumoral pathways of clonal evolution. *Histopathology* **2011**, *58*, 486-497.
5. Biankin, A.V.; Waddell, N.; Kassahn, K.S.; Gingras, M.C.; Muthuswamy, L.B.; Johns, A.L.; Miller, D.K.; Wilson, P.J.; Patch, A.M.; Wu, J.; et al. Pancreatic cancer genomes reveal aberrations in axon guidance pathway genes. *Nature* **2012**, *491*, 399-405.
6. Donahue, T.R.; Tran, L.M.; Hill, R.; Li, Y.; Kovochich, A.; Calvopina, J.H.; Patel, S.G.; Wu, N.; Hindoyan, A.; Farrell, J.J.; et al. Integrative survival-based molecular profiling of human pancreatic cancer. *Clin.Cancer Res.* **2012**, *18*, 1352-1363.
7. Winter, C.; Kristiansen, G.; Kersting, S.; Roy, J.; Aust, D.; Knosel, T.; Rummele, P.; Jahnke, B.; Hentrich, V.; Ruckert, F.; et al. Google goes cancer: improving outcome prediction for cancer patients by network-based ranking of marker genes. *PLoS Comput Biol* **2012**, *8*, e1002511, doi:10.1371/journal.pcbi.100251
8. Yachida, S.; White, C.M.; Naito, Y.; Zhong, Y.; Brosnan, J.A.; Macgregor-Das, A.M.; Morgan, R.A.; Saunders, T.; Laheru, D.A.; Herman, J.M.; et al. Clinical significance of the genetic landscape of pancreatic cancer and implications for identification of potential long-term survivors. *Clin.Cancer Res.* **2012**, *18*, 6339-6347.
9. Gutierrez, M.L.; Munoz-Bellvis, L.; Sarasquete, M.E.; Hernandez-Mejia, D.G.; Abad Mdel, M.; Bengoechea, O.; Corchete, L.; Gonzalez-Gonzalez, M.; Garcia-Garcia, J.; Gonzalez, M.; et al. Altered interphase fluorescence in situ hybridization profiles of chromosomes 4, 8q24, and 9q34 in pancreatic ductal adenocarcinoma are associated with a poorer patient outcome. *J Mol Diagn* **2014**, *16*, 648-659, doi:10.1016/j.jmoldx.2014.06.007.
10. Haider, S.; Wang, J.; Nagano, A.; Desai, A.; Arumugam, P.; Dumartin, L.; Fitzgibbon, J.; Hagemann, T.; Marshall, J.F.; Kocher, H.M.; et al. A multi-gene signature predicts outcome in patients with pancreatic ductal adenocarcinoma. *Genome medicine* **2014**, *6*, 105, doi:10.1186/s13073-014-0105-3.
11. Nones, K.; Waddell, N.; Song, S.; Patch, A.M.; Miller, D.; Johns, A.; Wu, J.; Kassahn, K.S.; Wood, D.; Bailey, P.; et al. Genome-wide DNA methylation patterns in pancreatic ductal adenocarcinoma reveal epigenetic deregulation of SLIT-ROBO, ITGA2 and MET signaling. *Int J Cancer* **2014**, *135*, 1110-1118, doi:10.1002/ijc.28765.
12. Dal Molin, M.; Zhang, M.; de Wilde, R.F.; Ottenhof, N.A.; Rezaee, N.; Wolfgang, C.L.; Blackford, A.; Vogelstein, B.; Kinzler, K.W.; Papadopoulos, N.; et al. Very Long-term Survival Following Resection for Pancreatic Cancer Is Not Explained by Commonly Mutated Genes: Results of Whole-Exome Sequencing Analysis. *Clin Cancer Res* **2015**, doi:10.1158/1078-0432.CCR-14-2600.
13. Namkung, J.; Kwon, W.; Choi, Y.; Yi, S.G.; Han, S.; Kang, M.J.; Kim, S.W.; Park, T.; Jang, J.Y. Molecular subtypes of pancreatic cancer based on miRNA expression profiles have independent prognostic value. *J Gastroenterol Hepatol* **2016**, *31*, 1160-1167, doi:10.1111/jgh.13253.
14. Schlitter, A.M.; Segler, A.; Steiger, K.; Michalski, C.W.; Jager, C.; Konukiewitz, B.; Pfarr, N.; Endris, V.; Bettstetter, M.; Kong, B.; et al. Molecular, morphological and survival analysis of 177 resected pancreatic ductal adenocarcinomas (PDACs): Identification of prognostic subtypes. *Scientific reports* **2017**, *7*, 41064, doi:10.1038/srep41064.
15. Lowery, M.A.; Kelsen, D.P.; Stadler, Z.K.; Yu, K.H.; Janjigian, Y.Y.; Ludwig, E.; D'Adamo, D.R.; Salo-Mullen, E.; Robson, M.E.; Allen, P.J.; et al. An emerging entity: pancreatic adenocarcinoma associated with a known BRCA mutation: clinical descriptors, treatment implications, and future directions. *Oncologist.* **2011**, *16*, 1397-1402.
16. Aung, K.L.; Fischer, S.E.; Denroche, R.E.; Jang, G.H.; Dodd, A.; Creighton, S.; Southwood, B.; Liang, S.B.; Chadwick, D.; Zhang, A.; et al. Genomics-Driven Precision Medicine for Advanced Pancreatic Cancer: Early Results from the COMPASS Trial. *Clin Cancer Res* **2018**, *24*, 1344-1354, doi:10.1158/1078-0432.CCR-17-2994.
17. Collisson, E.A.; Sadanandam, A.; Olson, P.; Gibb, W.J.; Truitt, M.; Gu, S.; Cooc, J.; Weinkle, J.; Kim, G.E.; Jakkula, L.; et al. Subtypes of pancreatic ductal adenocarcinoma and their differing responses to therapy. *Nat Med* **2011**, *17*, 500-503, doi:10.1038/nm.2344.
18. Mirzoeva, O.K.; Collisson, E.A.; Schaefer, P.M.; Hann, B.; Hom, Y.K.; Ko, A.H.; Korn, W.M. Subtype-specific MEK-PI3 kinase feedback as a therapeutic target in pancreatic adenocarcinoma. *Mol Cancer Ther* **2013**, *12*, 2213-2225, doi:10.1158/1535-7163.MCT-13-0104.

19. Kim, S.; Kang, M.; Lee, S.; Bae, S.; Han, S.; Jang, J.Y.; Park, T. Identifying molecular subtypes related to clinicopathologic factors in pancreatic cancer. *Biomed Eng Online* **2014**, *13 Suppl 2*, S5, doi:10.1186/1475-925X-13-S2-S5.
20. Daemen, A.; Peterson, D.; Sahu, N.; McCord, R.; Du, X.; Liu, B.; Kowanetz, K.; Hong, R.; Moffat, J.; Gao, M.; et al. Metabolite profiling stratifies pancreatic ductal adenocarcinomas into subtypes with distinct sensitivities to metabolic inhibitors. *Proceedings of the National Academy of Sciences of the United States of America* **2015**, *112*, E4410-4417, doi:10.1073/pnas.1501605112.
21. Galvan, J.A.; Zlobec, I.; Wartenberg, M.; Lugli, A.; Gloor, B.; Perren, A.; Karamitopoulou, E. Expression of E-cadherin repressors SNAIL, ZEB1 and ZEB2 by tumour and stromal cells influences tumour-budding phenotype and suggests heterogeneity of stromal cells in pancreatic cancer. *Br J Cancer* **2015**, *112*, 1944-1950, doi:10.1038/bjc.2015.177.
22. Gutierrez, M.L.; Corchete, L.; Teodosio, C.; Sarasquete, M.E.; del Mar Abad, M.; Iglesias, M.; Esteban, C.; Sayagues, J.M.; Orfao, A.; Munoz-Bellvis, L. Identification and characterization of the gene expression profiles for protein coding and non-coding RNAs of pancreatic ductal adenocarcinomas. *Oncotarget* **2015**, *6*, 19070-19086, doi:10.18632/oncotarget.4233.
23. Moffitt, R.A.; Marayati, R.; Flate, E.L.; Volmar, K.E.; Loeza, S.G.; Hoadley, K.A.; Rashid, N.U.; Williams, L.A.; Eaton, S.C.; Chung, A.H.; et al. Virtual microdissection identifies distinct tumor- and stroma-specific subtypes of pancreatic ductal adenocarcinoma. *Nat Genet* **2015**, *47*, 1168-1178, doi:10.1038/ng.3398.
24. Waddell, N.; Pajic, M.; Patch, A.M.; Chang, D.K.; Kassahn, K.S.; Bailey, P.; Johns, A.L.; Miller, D.; Nones, K.; Quek, K.; et al. Whole genomes redefine the mutational landscape of pancreatic cancer. *Nature* **2015**, *518*, 495-501, doi:10.1038/nature14169.
25. Bailey, P.; Chang, D.K.; Nones, K.; Johns, A.L.; Patch, A.M.; Gingras, M.C.; Miller, D.K.; Christ, A.N.; Bruxner, T.J.; Quinn, M.C.; et al. Genomic analyses identify molecular subtypes of pancreatic cancer. *Nature* **2016**, *531*, 47-52, doi:10.1038/nature16965.
26. Janky, R.; Binda, M.M.; Allemeersch, J.; Van den Broeck, A.; Govaere, O.; Swinnen, J.V.; Roskams, T.; Aerts, S.; Topal, B. Prognostic relevance of molecular subtypes and master regulators in pancreatic ductal adenocarcinoma. *BMC Cancer* **2016**, *16*, 632, doi:10.1186/s12885-016-2540-6.
27. Noll, E.M.; Eisen, C.; Stenzinger, A.; Espinet, E.; Muckenhuber, A.; Klein, C.; Vogel, V.; Klaus, B.; Nadler, W.; Rosli, C.; et al. CYP3A5 mediates basal and acquired therapy resistance in different subtypes of pancreatic ductal adenocarcinoma. *Nat Med* **2016**, *22*, 278-287, doi:10.1038/nm.4038.
28. Birnbaum, D.J.; Finetti, P.; Birnbaum, D.; Mamessier, E.; Bertucci, F. Validation and comparison of the molecular classifications of pancreatic carcinomas. *Mol Cancer* **2017**, *16*, 168, doi:10.1186/s12943-017-0739-z.
29. Connor, A.A.; Denroche, R.E.; Jang, G.H.; Timms, L.; Kalimuthu, S.N.; Selander, I.; McPherson, T.; Wilson, G.W.; Chan-Seng-Yue, M.A.; Borozan, I.; et al. Association of Distinct Mutational Signatures With Correlates of Increased Immune Activity in Pancreatic Ductal Adenocarcinoma. *JAMA Oncol* **2017**, *3*, 774-783, doi:10.1001/jamaoncol.2016.3916.
30. Knudsen, E.S.; Vail, P.; Balaji, U.; Ngo, H.; Botros, I.W.; Makarov, V.; Riaz, N.; Balachandran, V.; Leach, S.; Thompson, D.M.; et al. Stratification of Pancreatic Ductal Adenocarcinoma: Combinatorial Genetic, Stromal, and Immunologic Markers. *Clin Cancer Res* **2017**, *23*, 4429-4440, doi:10.1158/1078-0432.CCR-17-0162.
31. Kuhlmann, L.; Nadler, W.M.; Kerner, A.; Hanke, S.A.; Noll, E.M.; Eisen, C.; Espinet, E.; Vogel, V.; Trumpp, A.; Sprick, M.R.; et al. Identification and Validation of Novel Subtype-Specific Protein Biomarkers in Pancreatic Ductal Adenocarcinoma. *Pancreas* **2017**, *46*, 311-322, doi:10.1097/MPA.0000000000000743.
32. Nicolle, R.; Blum, Y.; Marisa, L.; Loncle, C.; Gayet, O.; Moutardier, V.; Turrini, O.; Giovannini, M.; Bian, B.; Bigonnet, M.; et al. Pancreatic Adenocarcinoma Therapeutic Targets Revealed by Tumor-Stroma Cross-Talk Analyses in Patient-Derived Xenografts. *Cell reports* **2017**, *21*, 2458-2470, doi:10.1016/j.celrep.2017.11.003.
33. Ohlund, D.; Handly-Santana, A.; Biffi, G.; Elyada, E.; Almeida, A.S.; Ponz-Sarvise, M.; Corbo, V.; Oni, T.E.; Hearn, S.A.; Lee, E.J.; et al. Distinct populations of inflammatory fibroblasts and myofibroblasts in pancreatic cancer. *J Exp Med* **2017**, *214*, 579-596, doi:10.1084/jem.20162024.
34. Cancer Genome Atlas Research Network. Electronic address, a.a.d.h.e.; Cancer Genome Atlas Research, N. Integrated Genomic Characterization of Pancreatic Ductal Adenocarcinoma. *Cancer cell* **2017**, *32*, 185-203 e113, doi:10.1016/j.ccell.2017.07.007.
35. Sivakumar, S.; de Santiago, I.; Chlon, L.; Markowetz, F. Master Regulators of Oncogenic KRAS Response in Pancreatic Cancer: An Integrative Network Biology Analysis. *PLoS Med* **2017**, *14*, e1002223, doi:10.1371/journal.pmed.1002223.
36. Dreyer, S.B.; Jamieson, N.B.; Upstill-Goddard, R.; Bailey, P.J.; McKay, C.J.; Australian Pancreatic Cancer Genome, I.; Biankin, A.V.; Chang, D.K. Defining the molecular pathology of pancreatic body and tail adenocarcinoma. *Br J Surg* **2018**, *105*, e183-e191, doi:10.1002/bjs.10772.
37. Erkan, M.; Michalski, C.W.; Rieder, S.; Reiser-Erkan, C.; Abiatari, I.; Kolb, A.; Giese, N.A.; Esposito, I.; Friess, H.; Kleeff, J. The activated stroma index is a novel and independent prognostic marker in pancreatic ductal adenocarcinoma. *Clin Gastroenterol Hepatol* **2008**, *6*, 1155-1161, doi:10.1016/j.cgh.2008.05.006.

38. Lomberk, G.; Blum, Y.; Nicolle, R.; Nair, A.; Gaonkar, K.S.; Marisa, L.; Mathison, A.; Sun, Z.; Yan, H.; Elarouci, N.; et al. Distinct epigenetic landscapes underlie the pathobiology of pancreatic cancer subtypes. *Nat Commun* **2018**, *9*, 1978, doi:10.1038/s41467-018-04383-6.
39. Muckenhuber, A.; Berger, A.K.; Schlitter, A.M.; Steiger, K.; Konukiewitz, B.; Trumpp, A.; Eils, R.; Werner, J.; Friess, H.; Esposito, I.; et al. Pancreatic Ductal Adenocarcinoma Subtyping Using the Biomarkers Hepatocyte Nuclear Factor-1A and Cytokeratin-81 Correlates with Outcome and Treatment Response. *Clin Cancer Res* **2018**, *24*, 351-359, doi:10.1158/1078-0432.CCR-17-2180.
40. Puleo, F.; Nicolle, R.; Blum, Y.; Cros, J.; Marisa, L.; Demetter, P.; Quertinmont, E.; Svrcek, M.; Elarouci, N.; Iovanna, J.; et al. Stratification of Pancreatic Ductal Adenocarcinomas Based on Tumor and Microenvironment Features. *Gastroenterology* **2018**, *155*, 1999-2013 e1993, doi:10.1053/j.gastro.2018.08.033.
41. Wartenberg, M.; Cibin, S.; Zlobec, I.; Vassella, E.; Eppenberger-Castori, S.; Terracciano, L.; Eichmann, M.D.; Worni, M.; Gloor, B.; Perren, A.; et al. Integrated Genomic and Immunophenotypic Classification of Pancreatic Cancer Reveals Three Distinct Subtypes with Prognostic/Predictive Significance. *Clin Cancer Res* **2018**, *24*, 4444-4454, doi:10.1158/1078-0432.CCR-17-3401.
42. Zhao, L.; Zhao, H.; Yan, H. Gene expression profiling of 1200 pancreatic ductal adenocarcinoma reveals novel subtypes. *BMC Cancer* **2018**, *18*, 603, doi:10.1186/s12885-018-4546-8.
43. Birnbaum, D.J.; Bertucci, F.; Finetti, P.; Birnbaum, D.; Mamessier, E. Head and Body/Tail Pancreatic Carcinomas Are Not the Same Tumors. *Cancers (Basel)* **2019**, *11*, doi:10.3390/cancers11040497.
44. Connor, A.A.; Denroche, R.E.; Jang, G.H.; Lemire, M.; Zhang, A.; Chan-Seng-Yue, M.; Wilson, G.; Grant, R.C.; Merico, D.; Lungu, I.; et al. Integration of Genomic and Transcriptional Features in Pancreatic Cancer Reveals Increased Cell Cycle Progression in Metastases. *Cancer cell* **2019**, *35*, 267-282 e267, doi:10.1016/j.ccell.2018.12.010.
45. de Santiago, I.; Yau, C.; Heij, L.; Middleton, M.R.; Markowetz, F.; Grabsch, H.I.; Dustin, M.L.; Sivakumar, S. Immunophenotypes of pancreatic ductal adenocarcinoma: Meta-analysis of transcriptional subtypes. *Int J Cancer* **2019**, *145*, 1125-1137, doi:10.1002/ijc.32186.
46. Dijk, F.; Veenstra, V.L.; Soer, E.C.; Dings, M.P.G.; Zhao, L.; Halfwerk, J.B.; Hooijer, G.K.; Damhofer, H.; Marzano, M.; Steins, A.; et al. Unsupervised class discovery in pancreatic ductal adenocarcinoma reveals cell-intrinsic mesenchymal features and high concordance between existing classification systems. *Scientific reports* **2020**, *10*, 337, doi:10.1038/s41598-019-56826-9.
47. Elyada, E.; Bolisetty, M.; Laise, P.; Flynn, W.F.; Courtois, E.T.; Burkhart, R.A.; Teinor, J.A.; Belleau, P.; Biffi, G.; Lucito, M.S.; et al. Cross-Species Single-Cell Analysis of Pancreatic Ductal Adenocarcinoma Reveals Antigen-Presenting Cancer-Associated Fibroblasts. *Cancer Discov* **2019**, *9*, 1102-1123, doi:10.1158/2159-8290.CD-19-0094.
48. Follia, L.; Ferrero, G.; Mandili, G.; Beccuti, M.; Giordano, D.; Spadi, R.; Satolli, M.A.; Evangelista, A.; Katayama, H.; Hong, W.; et al. Integrative Analysis of Novel Metabolic Subtypes in Pancreatic Cancer Fosters New Prognostic Biomarkers. *Front Oncol* **2019**, *9*, 115, doi:10.3389/fonc.2019.00115.
49. Hennig, A.; Wolf, L.; Jahnke, B.; Polster, H.; Seidlitz, T.; Werner, K.; Aust, D.E.; Hampe, J.; Distler, M.; Weitz, J.; et al. CFTR Expression Analysis for Subtyping of Human Pancreatic Cancer Organoids. *Stem Cells Int* **2019**, *2019*, 1024614, doi:10.1155/2019/1024614.
50. SN, K.; Wilson, G.W.; Grant, R.C.; Seto, M.; O'Kane, G.; Vajpeyi, R.; Notta, F.; Gallinger, S.; Chetty, R. Morphological classification of pancreatic ductal adenocarcinoma that predicts molecular subtypes and correlates with clinical outcome. *Gut* **2020**, *69*, 317-328, doi:10.1136/gutjnl-2019-318217.
51. Ligorio, M.; Sil, S.; Malagon-Lopez, J.; Nieman, L.T.; Misale, S.; Di Pilato, M.; Ebright, R.Y.; Karabacak, M.N.; Kulkarni, A.S.; Liu, A.; et al. Stromal Microenvironment Shapes the Intratumoral Architecture of Pancreatic Cancer. *Cell* **2019**, *178*, 160-175 e127, doi:10.1016/j.cell.2019.05.012.
52. Maurer, C.; Holmstrom, S.R.; He, J.; Laise, P.; Su, T.; Ahmed, A.; Hibshoosh, H.; Chabot, J.A.; Oberstein, P.E.; Sepulveda, A.R.; et al. Experimental microdissection enables functional harmonisation of pancreatic cancer subtypes. *Gut* **2019**, *68*, 1034-1043, doi:10.1136/gutjnl-2018-317706.
53. Neuzillet, C.; Tijeras-Raballand, A.; Ragulan, C.; Cros, J.; Patil, Y.; Martinet, M.; Erkan, M.; Kleeff, J.; Wilson, J.; Apte, M.; et al. Inter- and intra-tumoural heterogeneity in cancer-associated fibroblasts of human pancreatic ductal adenocarcinoma. *J Pathol* **2019**, *248*, 51-65, doi:10.1002/path.5224.
54. Rashid, N.U.; Peng, X.L.; Jin, C.; Moffitt, R.A.; Volmar, K.E.; Belt, B.A.; Panni, R.Z.; Nywening, T.M.; Herrera, S.G.; Moore, K.J.; et al. Purity Independent Subtyping of Tumors (PurIST), A Clinically Robust, Single-sample Classifier for Tumor Subtyping in Pancreatic Cancer. *Clin Cancer Res* **2020**, *26*, 82-92, doi:10.1158/1078-0432.CCR-19-1467.
55. Brunton, H.; Caligiuri, G.; Cunningham, R.; Upstill-Goddard, R.; Bailey, U.M.; Garner, I.M.; Nourse, C.; Dreyer, S.; Jones, M.; Moran-Jones, K.; et al. HNF4A and GATA6 Loss Reveals Therapeutically Actionable Subtypes in Pancreatic Cancer. *Cell reports* **2020**, *31*, 107625, doi:10.1016/j.celrep.2020.107625.

56. Demirkol Canli, S.; Dedeoglu, E.; Akbar, M.W.; Kucukkaraduman, B.; Isbilen, M.; Erdogan, O.S.; Erciyas, S.K.; Yazici, H.; Vural, B.; Gure, A.O. A novel 20-gene prognostic score in pancreatic adenocarcinoma. *PLoS One* **2020**, *15*, e0231835, doi:10.1371/journal.pone.0231835.
57. Chan-Seng-Yue, M.; Kim, J.C.; Wilson, G.W.; Ng, K.; Figueroa, E.F.; O'Kane, G.M.; Connor, A.A.; Denroche, R.E.; Grant, R.C.; McLeod, J.; et al. Transcription phenotypes of pancreatic cancer are driven by genomic events during tumor evolution. *Nat Genet* **2020**, *52*, 231-240, doi:10.1038/s41588-019-0566-9.
58. Glass, M.; Dorn, A.; Huttelmaier, S.; Haemmerle, M.; Gutschner, T. Comprehensive Analysis of LincRNAs in Classical and Basal-Like Subtypes of Pancreatic Cancer. *Cancers (Basel)* **2020**, *12*, doi:10.3390/cancers12082077.
59. Hayashi, A.; Fan, J.; Chen, R.; Ho, Y.-j.; Makohon-Moore, A.P.; Lecomte, N.; Zhong, Y.; Hong, J.; Huang, J.; Sakamoto, H.; et al. A unifying paradigm for transcriptional heterogeneity and squamous features in pancreatic ductal adenocarcinoma. *Nature Cancer* **2020**, *1*, 59-74, doi:10.1038/s43018-019-0010-1.
60. Juiz, N.; Elkaoutari, A.; Bigonnet, M.; Gayet, O.; Roques, J.; Nicolle, R.; Iovanna, J.; Dusetti, N. Basal-like and classical cells coexist in pancreatic cancer revealed by single-cell analysis on biopsy-derived pancreatic cancer organoids from the classical subtype. *FASEB J* **2020**, *34*, 12214-12228, doi:10.1096/fj.202000363RR.
61. Kalloger, S.E.; Karasinska, J.M.; Keung, M.S.; Thompson, D.L.; Ho, J.; Chow, C.; Gao, D.; Topham, J.T.; Warren, C.; Wong, H.L.; et al. Stroma vs epithelium-enhanced prognostics through histologic stratification in pancreatic ductal adenocarcinoma. *Int J Cancer* **2021**, *148*, 481-491, doi:10.1002/ijc.33304.
62. Kandimalla, R.; Tomihara, H.; Banwait, J.K.; Yamamura, K.; Singh, G.; Baba, H.; Goel, A. A 15-Gene Immune, Stromal, and Proliferation Gene Signature that Significantly Associates with Poor Survival in Patients with Pancreatic Ductal Adenocarcinoma. *Clin Cancer Res* **2020**, *26*, 3641-3648, doi:10.1158/1078-0432.CCR-19-4044.
63. Karasinska, J.M.; Topham, J.T.; Kalloger, S.E.; Jang, G.H.; Denroche, R.E.; Culibrk, L.; Williamson, L.M.; Wong, H.L.; Lee, M.K.C.; O'Kane, G.M.; et al. Altered Gene Expression along the Glycolysis-Cholesterol Synthesis Axis Is Associated with Outcome in Pancreatic Cancer. *Clin Cancer Res* **2020**, *26*, 135-146, doi:10.1158/1078-0432.CCR-19-1543.
64. Kaissis, G.A.; Ziegelmayer, S.; Lohofer, F.K.; Harder, F.N.; Jungmann, F.; Sasse, D.; Muckenhuber, A.; Yen, H.Y.; Steiger, K.; Siveke, J.; et al. Image-Based Molecular Phenotyping of Pancreatic Ductal Adenocarcinoma. *J Clin Med* **2020**, *9*, doi:10.3390/jcm9030724.
65. Lin, W.; Noel, P.; Borazanci, E.H.; Lee, J.; Amini, A.; Han, I.W.; Heo, J.S.; Jameson, G.S.; Fraser, C.; Steinbach, M.; et al. Single-cell transcriptome analysis of tumor and stromal compartments of pancreatic ductal adenocarcinoma primary tumors and metastatic lesions. *Genome medicine* **2020**, *12*, 80, doi:10.1186/s13073-020-00776-9.
66. Topham, J.T.; Karasinska, J.M.; Lee, M.K.C.; Csizmok, V.; Williamson, L.M.; Jang, G.H.; Denroche, R.E.; Tsang, E.S.; Kalloger, S.E.; Wong, H.L.; et al. Subtype-Discordant Pancreatic Ductal Adenocarcinoma Tumors Show Intermediate Clinical and Molecular Characteristics. *Clin Cancer Res* **2021**, *27*, 150-157, doi:10.1158/1078-0432.CCR-20-2831.
67. Zhang, Y.; Zhu, L.; Wang, X. A Network-Based Approach for Identification of Subtype-Specific Master Regulators in Pancreatic Ductal Adenocarcinoma. *Genes (Basel)* **2020**, *11*, doi:10.3390/genes11020155.
